# USP9X counteracts differential ubiquitination of NPHP5 by MARCH7 and BBS11 to regulate ciliogenesis

**DOI:** 10.1371/journal.pgen.1006791

**Published:** 2017-05-12

**Authors:** Arindam Das, Jin Qian, William Y. Tsang

**Affiliations:** 1 Institut de recherches cliniques de Montréal, Montréal, Québec, Canada; 2 Faculté de Médecine, Département de pathologie et biologie cellulaire, Université de Montréal, Montréal, Québec, Canada; 3 Division of Experimental Medicine, McGill University, Montréal, Québec, Canada; Washington University School of Medicine, UNITED STATES

## Abstract

Ciliogenesis is a fundamental biological process central to human health. Precisely how this process is coordinated with the cell cycle remains an open question. We report that nephrocystin-5 (NPHP5/IQCB1), a positive regulator of ciliogenesis, is a stable and low turnover protein subjected to cycles of ubiquitination and deubiquitination. NPHP5 directly binds to a deubiquitinating enzyme USP9X/FAM and two E3 ubiquitin ligases BBS11/TRIM32 and MARCH7/axotrophin. NPHP5 undergoes K63 ubiquitination in a cell cycle dependent manner and K48/K63 ubiquitination upon USP9X depletion or inhibition. In the G0/G1/S phase, a pool of cytoplasmic USP9X recruited to the centrosome by NPHP5 protects NPHP5 from ubiquitination, thus favouring cilia assembly. In the G2/M phase, USP9X dissociation from the centrosome allows BBS11 to K63 ubiquitinate NPHP5 which triggers protein delocalization and loss of cilia. BBS11 is a resident centrosomal protein, whereas cytoplasmic USP9X sequesters the majority of MARCH7 away from the centrosome during interphase. Depletion or inhibition of USP9X leads to an accumulation of centrosomal MARCH7 which K48 ubiquitinates NPHP5, triggering protein degradation and cilia loss. At the same time, BBS11 K63 ubiquitinates NPHP5. Our data suggest that dynamic ubiquitination and deubiquitination of NPHP5 plays a crucial role in the regulation of ciliogenesis.

## Author summary

Centrosomes are non-membrane bound organelles that modulate a variety of cellular processes including cell division and formation of hair-like protrusions called primary cilia. Primary cilia function as cellular antennae to sense a wide variety of signals important for growth, development and differentiation. Defects in cilia formation or ciliogenesis can give rise to a bewildering array of human ciliary diseases collectively known as ciliopathies. Ciliogenesis is controlled in part by nephrocystin-5 (NPHP5/IQCB1), and NPHP5 dysfunction causes ciliopathies in humans, mice and dogs. We are interested in studying how the stability, localization and biological activity of NPHP5 are regulated at the molecular level. We present here that NPHP5 directly interacts with, and is a substrate of, one deubiquitinase (USP9X/FAM) and two ubiquitin ligases (BBS11/TRIM32 and MARCH7/axotrophin), enzymes involved in controlling protein stability, localization and activity. Our results suggest that timely ubiquitination and deubiquitination of NPHP5 is critical for the regulation of ciliogenesis.

## Introduction

Primary cilia, microtubule-based protrusions found on the surface of most eukaryotic cells, are derived from centrosomes and possess sensory function such as chemosensation and mechanosensation[1,2]. Formation of primary cilia is tightly regulated during the cell cycle: they assemble primarily during the G0 phase and undergo complete disassembly prior to entry into mitosis[3]. Defects in cilia formation (ciliogenesis) or function can give rise to a myriad of human genetic disorders collectively known as ciliopathies that are often pleiotropic, exhibiting clinical manifestations such as retinal degeneration, renal failure and neurological disorders[4]. In addition, cilia regulate several signalling pathways commonly perturbed in cancer and a loss of cilia is known to occur early in the development of several human cancers[5,6,7,8,9,10,11,12,13,14]. Although several hundred proteins are required for ciliogenesis[15,16,17], a critical step towards understanding their role in health and disease is to delineate their precise spatial and temporal regulation.

Ciliogenesis is controlled in part by nephrocystin-5 (NPHP5/IQCB1). *NPHP5* was originally identified as the causative gene of two human ciliopathies, Senior-Løken syndrome and Leber congenital amaurosis, typified by retinal degeneration with or without renal failure[18,19,20]. Murine and canine models of NPHP5 develop retinal degeneration[21,22]. NPHP5 might also be involved in tumorigenesis since its mRNA expression is up-regulated in gastrointestinal cancer[23]. We are others have shown that NPHP5 and its interacting partner Cep290 are essential for ciliogenesis[24,25]. Pathogenic mutations of *NPHP5* lead to truncated products that become mis-localized and are unable to interact with Cep290[25]. NPHP5 localizes to the centrosome including the ciliary base during interphase[24,25] but disappears from the organelle during mitosis for reasons that are not understood[25]. Moreover, exactly how the stability or activity of this protein is controlled at the molecular level has not been studied.

Ubiquitination is a post-translational modification crucial for controlling protein stability, localization and activity[26]. It is a multi-step process in which ubiquitin (Ub) is transferred onto a substrate via the action of three enzymes: an Ub-activating enzyme E1, an Ub-conjugating enzyme E2 and an Ub ligase E3 which is mainly responsible for substrate recognition. In humans, several hundred E3 ligases exist and they are grouped into three families based on the presence of characteristic domains and the mechanism of ubiquitin transfer[27]. A given substrate can be monoubiquitinated, multi-monoubiquitinated or polyubiquitinated. The most common types of polyubiquitination are the K48-linkage which targets a substrate for proteasomal degradation, and the K63-linkage which has non-proteasomal function. Substrate ubiquitination by E3 ligases can be reversed by the ~100 or so deubiquitinating enzymes or deubiquitinases (DUBs) that are divided into five families[28,29]. E3 ligases often work in concert with DUBs to control the ubiquitination status of a substrate, and deregulation of these enzymes is known to cause human disorders and cancer[30,31]. It is currently unknown if NPHP5 associates with E3 ligases and/or DUBs and undergoes ubiquitination and/or deubiquitination.

## Results

### NPHP5 is polyubiquitinated in cells

**Fig 1:**
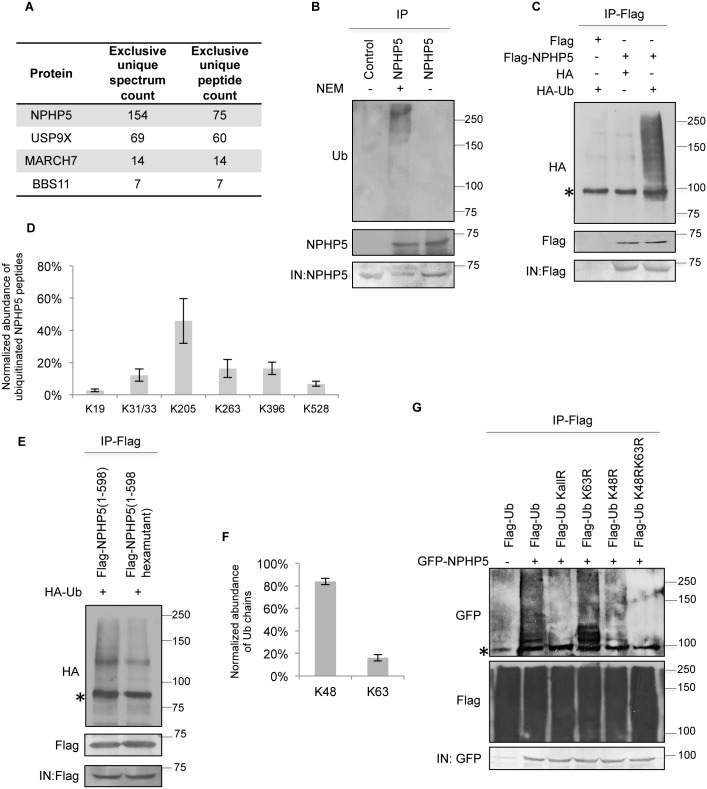
NPHP5 is polyubiquitinated in cells. **A**) Flag-NPHP5 complexes from HEK293 cells were immuno-purified and subjected to mass spectrometric analysis. **B)** HEK293 cell lysates treated with or without a pan DUB inhibitor NEM were immunoprecipitated with anti-IgG (control) or anti-NPHP5 antibody and Western blotted with the indicated antibodies. IN, input. **C)** HEK293 cells were transfected with Flag or Flag-NPHP5 and HA or HA-Ub. Lysates were immunoprecipitated with anti-Flag antibody in 1% SDS and Western blotted with the indicated antibodies. IN, input. **D**) Flag-NPHP5 expressed in HEK293 cells was immuo-purified in the presence of NEM. Mass spectrometric quantitation of NPHP5 ubiquitination sites is presented. **E**) HEK293 cells were transfected with HA-Ub and Flag-NPHP5 wild type or hexamutant (K31RK33RK205RK263RK396RK528R). Lysates were immunoprecipitated with anti-Flag antibody in 1% SDS and Western blotted with the indicated antibodies. IN, input. **F)** Flag-NPHP5 expressed in HEK293 cells was immuno-purified in the presence of NEM. Mass spectrometric quantitation of Ub chain types attached to NPHP5 is presented. **G)** GFP-NPHP5 was co-expressed with Flag-Ub wild type, single mutant (K48R or K63R), double mutant (K48RK63R) or mutant in which all lysine residues were mutated to arginines (KallR) in HEK293 cells. Lysates were NEM treated and immunoprecipitated with anti-Flag antibody in 1% SDS and Western blotted with the indicated antibodies. IN, input. In **D, F)**, data were obtained from two independent experiments. Asterisks indicate non-specific bands.

In an effort to identify factors that regulate NPHP5 and hence ciliogenesis, we performed a proteomic screen for components of NPHP5-containining complexes. Three enzymes involved in substrate ubiquitination and deubiquitination[32,33,34], including one DUB, USP9X/FAM, and two E3 ligases, MARCH7/axotrophin and BBS11/TRIM32, were found (Fig 1A and S1 Table). We first explored whether NPHP5 can undergo ubiquitination by conducting *in vivo* ubiquitination assays. Polyubiquitination of NPHP5 was detected upon immunoprecipitating endogenous protein from HEK293 cells treated with a pan DUB inhibitor N-ethylmaleimide (NEM[35]) (Fig 1B). When Flag-NPHP5 was immunoprecipitated under denaturing conditions with 1% SDS to prevent co-immunoprecipitation of interacting proteins such as Cep290 and Usp9x (S1A Fig), the recombinant protein remained highly polyubiquitinated in the presence of NEM (S1B Fig), suggesting that NPHP5 itself undergoes ubiquitination. Polyubiquitination of NPHP5 could be observed without NEM when immunoprecipitations were performed from lysates of HEK293 cells expressing recombinant Flag-NPHP5 and HA-Ub (Fig 1C and S2A Fig). Of note, NPHP5 polyubiquitination occurred in multiple cell lines including HEK293, U2OS and normal diploid retinal pigmented epithelial cells (RPE-1), a well-established model for primary cilia formation (Fig 1C and S2A–S2C Fig). To identify ubiquitination sites on NPHP5 and characterize Ub chain linkages, ubiquitinated NPHP5 species purified from asynchronous cell extracts treated with NEM were analyzed by mass spectrometry. A double glycine was attached to several lysine residues of NPHP5, including K19, K31/K33, K205, K263, K396 and K528, suggesting that these residues are potential ubiquitination sites (Fig 1D). Indeed, a NPHP5 mutant in which six of these lysine residues were mutated to arginines (K19RK31RK33RK205RK263RK528R or hexamutant) exhibited diminished levels of ubiquitination compared to wild type (Fig 1E). In addition, about 80% and 20% of double glycines were linked to K48 and K63 residues of Ub, respectively (Fig 1F), indicating that NPHP5 is conjugated to both K48- and K63-Ub chains. To validate this finding, we observed robust ubiquitination of NPHP5 in the presence of NEM when this protein was co-expressed with wild type Ub or K63R mutant Ub, but not with K48RK63R Ub or KallR Ub in which all lysine residues were mutated to arginine (Fig 1G). The use of K48R mutant Ub did not completely abolish NPHP5 ubiquitination (Fig 1G), suggesting that a fraction of NPHP5 is K63-ubiquitinated. Taken together, these data indicate that NPHP5 is ubiquitinated at multiple sites and undergoes K48 and K63 ubiquitination upon DUB inhibition.

### USP9X directly binds and deubiquitinates NPHP5

**Fig 2:**
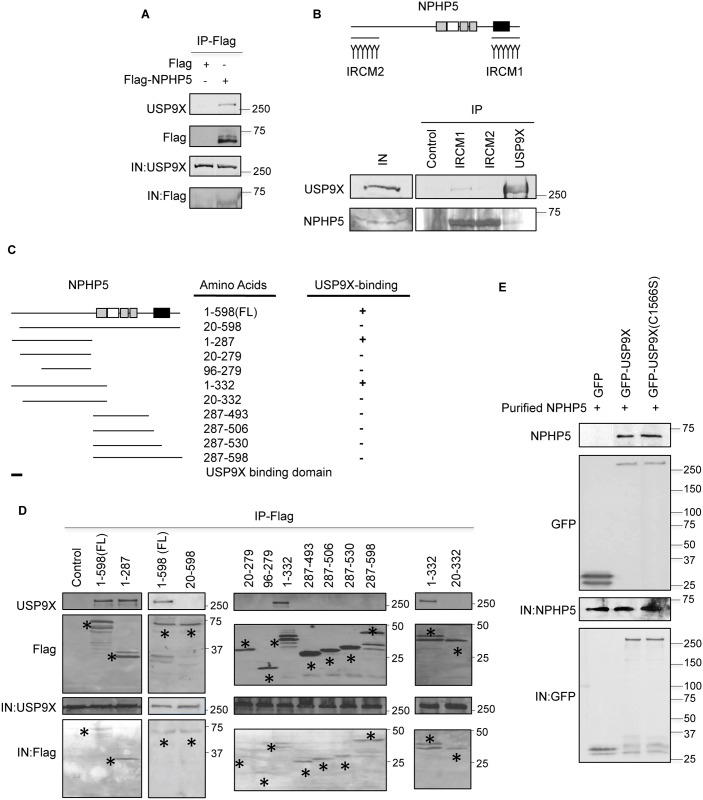
NPHP5 directly interacts with USP9X. **A)** Flag or Flag-NPHP5 was expressed in HEK293 cells. Lysates were immunoprecipitated with anti-Flag antibody and Western blotted with the indicated antibodies. IN, input. **B)** (Top) IRCM1 and IRCM2 are two anti-NPHP5 antibodies raised against the C- and the N-terminus of NPHP5, respectively. Grey box, calmodulin-binding domain; white box, coiled-coil domain; black box; Cep290-binding domain. (Bottom) HEK293 lysates were immunoprecipitated with anti-Flag (control), anti-NPHP5 (IRCM1 or IRCM2) or anti-USP9X antibody and Western blotted with the indicated antibodies. IN, input. **C)** The ability of full-length NPHP5 (1-598(FL)) and various NPHP5 truncates to interact with USP9X is presented. **D)** Flag (control) or the indicated fragment of Flag-tagged NPHP5 was expressed in HEK293 cells. Lysates were immunoprecipitated with anti-Flag antibody and Western blotted with the indicated antibodies. IN, input. Asterisks indicate bands corresponding to the expected recombinant proteins. **E)** Purified GFP, GFP-USP9X wild type or catalytically inactive mutant (C1566S) bound to beads was mixed with purified Flag-NPHP5. Proteins recovered on beads were analyzed by Western blotting with the indicated antibodies. IN, input.

Given that NPHP5 is ubiquitinated in cells and that a relatively large number of unique peptides corresponding to USP9X were identified in our proteomic screen (Fig 1A), we focused on verifying the interaction between NPHP5 and USP9X. We expressed Flag-NPHP5 in HEK293 cells, performed anti-Flag immunoprecipitations, and showed that recombinant NPHP5 and endogenous USP9X are co-precipitated (Fig 2A). Endogenous NPHP5 was detected in USP9X immunoprecipitates in HEK293 cells (Fig 2B) and similarly, endogenous USP9X was co-immunoprecipitated with an NPHP5 antibody against the C-terminus of the protein in HEK293, U2OS and RPE-1 cells (Fig 2B and see below). In sharp contrast, an NPHP5 antibody against the N-terminus of the protein did not efficiently immunoprecipitate USP9X (Fig 2B), suggesting that this antibody might compete with USP9X for binding to NPHP5. Indeed, mapping studies using a series of Flag-tagged NPHP5 truncates revealed that the N-terminal region spanning residues 1–19 is critical for USP9X binding (Fig 2C and 2D). The interaction between NPHP5 and USP9X is probably direct since purified NPHP5 bound to USP9X *in vitro* (Fig 2E). Both wild type and catalytically inactive mutant USP9X (C1566S) could bind to NPHP5 in cells and *in vitro* (Fig 2E and S3 Fig).

**Fig 3:**
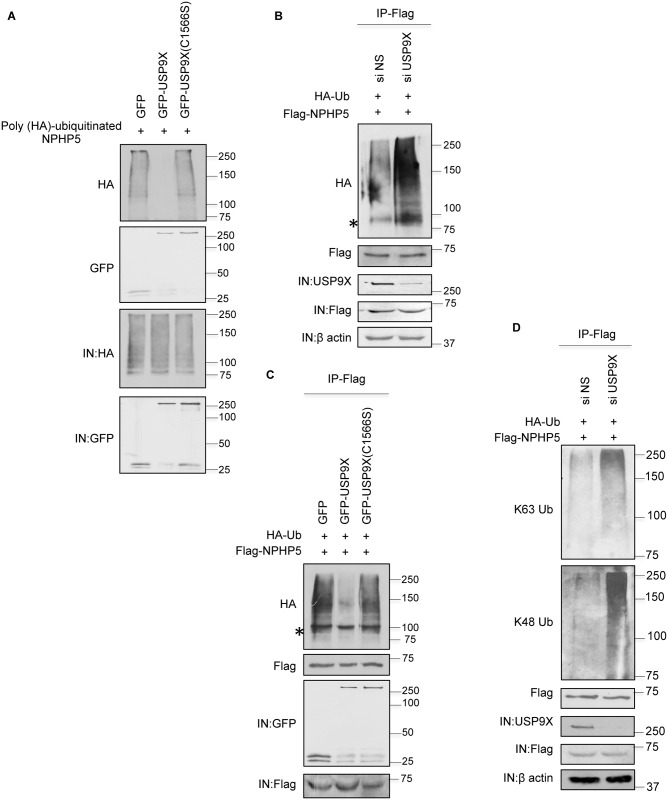
NPHP5 is deubiquitinated by USP9X. **A)** *In vitro* deubiquitination assays were performed by adding poly (HA)-ubiquitinated NPHP5 as a substrate to GFP, GFP-USP9X wild type or mutant (C1566S) bound to beads. Poly (HA)-ubiquitinated NPHP5 and GFP proteins in the reaction mixture were detected by immunoblotting with anti-HA and anti-GFP antibodies, respectively. IN, input. **B, D)** HEK293 cells were transfected with control (NS) or USP9X siRNA and plasmids expressing Flag-NPHP5 and HA-Ub. Lysates were subjected to immunoprecipitation with anti-Flag antibody in 1% SDS and Western blotted with the indicated antibodies. IN, input. **C)** HEK293 cells were transfected with GFP, GFP-USP9X wild type or mutant (C1566S), Flag-NPHP5 and HA-Ub. Lysates were immunoprecipitated with anti-Flag antibody in 1% SDS and Western blotted with the indicated antibodies. IN, input. Asterisks indicate non-specific bands.

To determine if NPHP5 is a substrate of USP9X, we performed *in vitro* deubiquitination experiments by incubating ubiquitinated NPHP5 with purified USP9X. Only wild type USP9X could robustly deubiquitinate NPHP5 (Fig 3A). Subsequently, we examined the effects of manipulating USP9X levels in cells, and showed that depletion of USP9X induces NPHP5 ubiquitination (Fig 3B), whereas expression of wild type but not mutant USP9X suppresses ubiquitination (Fig 3C). These results indicate that USP9X directly binds to, and catalyzes the deubiquitination of, NPHP5.

### NPHP5 becomes K48 and K63 ubiquitinated upon USP9X depletion/inhibition

**Fig 4:**
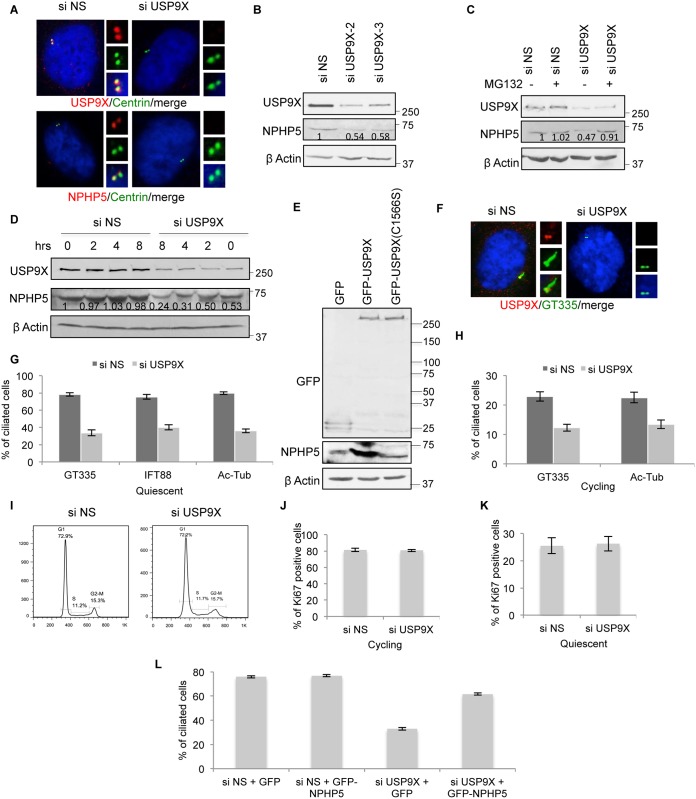
Effects of deregulating USP9X on NPHP5. **A)** RPE-1 cells transfected with control (NS) or USP9X siRNA and stained with antibodies against USP9X or NPHP5 (red) and centrin (green). DNA was stained with DAPI (blue). **B)** HEK293 cells were transfected with control (NS) or USP9X siRNA. Lysates were Western blotted with the indicated antibodies. β actin was used as a loading control. Numbers indicate NPHP5 protein levels relative to control. **C)** HEK293 cells were transfected with control (NS) or USP9X siRNA and treated with or without MG132. Lysates were Western blotted with the indicated antibodies. β actin was used as a loading control. Numbers indicate NPHP5 protein levels relative to control without MG132 treatment. **D)** HEK293 cells were transfected with control (NS) or USP9X siRNA and treated with cycloheximide for the indicated number of hours (hrs). Lysates were Western blotted with the indicated antibodies. β actin was used as a loading control. Numbers indicate NPHP5 protein levels relative to control at time zero. **E)** HEK293 cells were transfected with GFP, GFP-USP9X wild type or mutant (C1566S). Lysates were Western blotted with the indicated antibodies. β actin was used as a loading control. **F)** RPE-1 cells were transfected with control (NS) or USP9X siRNA, induced to quiescence, and stained with antibodies against USP9X (red) and glutamylated tubulin (GT335; green), and with DAPI (blue). **G-H)** The percentage of control (NS) or USP9X siRNA-treated cycling or quiescent RPE-1 cells with cilia was determined. Cilia were stained with glutamylated tubulin (GT335), acetylated tubulin (Ac-Tub) or IFT88. **I)** Fluorescence-activated cell sorting analysis of cycling RPE-1 cells treated with control (NS) or USP9X siRNA. **J-K**) The percentage of control (NS) or USP9X siRNA-treated RPE-1 cells stained positive for Ki67 was determined. **L)** RPE-1 cells transfected with control (NS) or USP9X siRNA and plasmid expressing GFP or GFP-NPHP5 were induced to quiescence. The percentage of GFP positive cells with cilia was determined. In **G, H, J, K, L)**, at least 100 cells were counted per condition, and error bars represent average of three independent experiments.

Next, we sought to characterize the nature and impact of NPHP5 ubiquitination. NPHP5 underwent K48 and K63 ubiquitination upon USP9X depletion (Fig 3D), akin to our earlier findings with DUB inhibition (Fig 1F and 1G). Depletion of USP9X significantly reduces the intensity of NPHP5 immunofluorescence at the centrosome (Fig 4A and S4 Fig) and protein levels, endogenous (Fig 4B and S5A Fig) or recombinant (S5B Fig), without affecting mRNA levels (S5C and S5D Fig). The decrease in NPHP5 protein levels could be rescued by a proteasome inhibitor MG132 (Fig 4C and S5E Fig) and enhanced by a protein synthesis inhibitor cycloheximide (Fig 4D and S5F Fig). Conversely, over-expression of wild type but not mutant USP9X elevated the protein levels of NPHP5 (Fig 4E). Ablation of USP9X inhibited the formation of cilia in cycling and quiescent RPE-1 cells (Fig 4F and 4H), a phenotype reminiscent of NPHP5 loss, without interfering with cell cycle progression (Fig 4I), cell proliferation (Fig 4J) or cell cycle exit (Fig 4K). Interestingly, cilia formation in USP9X-depleted cells could be restored by enforced expression of NPHP5 (Fig 4L). These observations together suggest that depletion/inhibition of USP9X sensitizes NPHP5 for K48 and K63 ubiquitination and proteasomal degradation, thereby compromising ciliogenesis.

### NPHP5 recruits USP9X to the centrosome

**Fig 5:**
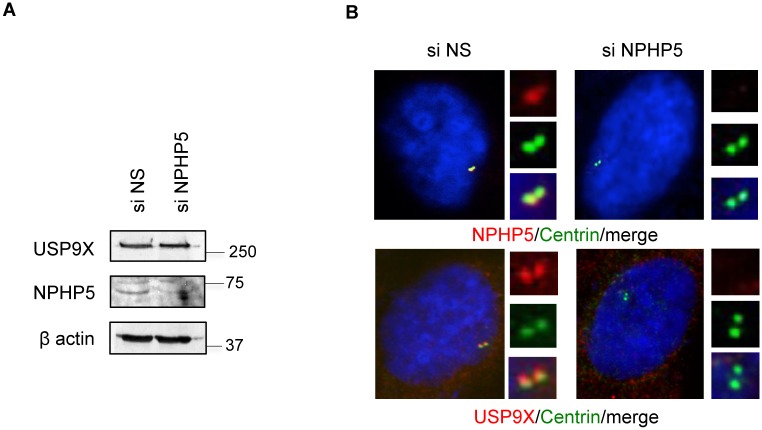
NPHP5 recruits USP9X to the centrosome. **A)** HEK293 cells were transfected with control (NS) and NPHP5 siRNA. Lysates were Western blotted with the indicated antibodies. β actin was used as a loading control. **B)** RPE-1 cells transfected with control (NS) and NPHP5 siRNA were stained with anti-NPHP5 or anti-USP9X (red) and anti-centrin (green) antibodies. DNA was stained with DAPI (blue).

**Fig 6:**
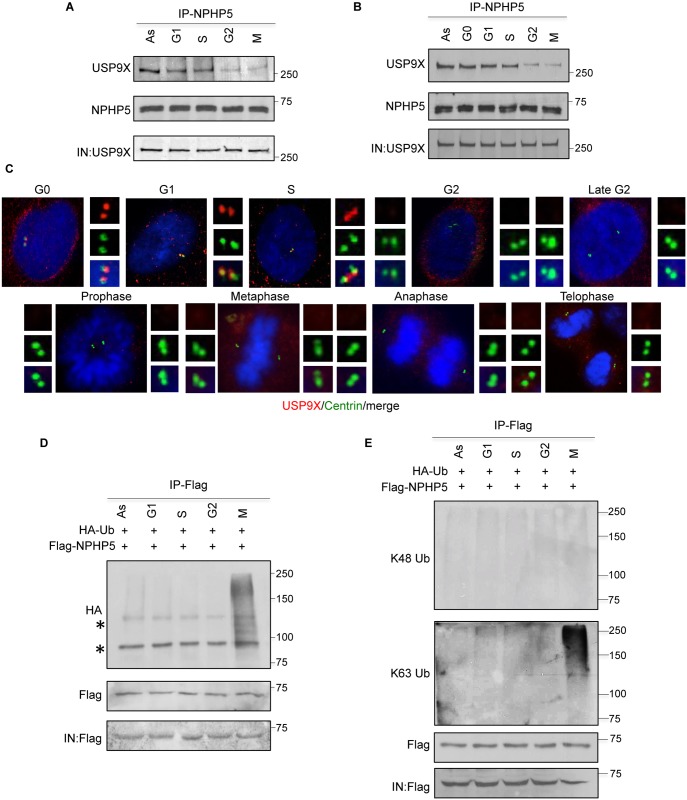
NPHP5 is a low turnover protein which becomes K63 ubiquitinated in mitosis. **A-B)** U2OS **A)** and RPE-1 **B)** lysates from different cell cycle phases were immunoprecipitated with anti-NPHP5 (IRCM1) antibody and Western blotted with the indicated antibodies. As, asynchronous; IN, input. **C)** RPE-1 cells in different stages of the cell cycle were processed for immunofluorescence with anti-USP9X (red) and anti-centrin (green) antibodies. DNA was stained with DAPI (blue). **D-E)** U2OS cells expressing Flag-NPHP5 and HA-Ub were synchronized in different cell cycle phases or left asynchronized (As). Lysates were immunoprecipitated with anti-Flag antibody in 1% SDS and Western blotted with the indicated antibodies. IN, input. Asterisks indicate non-specific bands.

To further explore the connection between NPHP5 and USP9X, we examined the consequences of ablating NPHP5 on USP9X protein levels and localization. Loss of NPHP5 did not impinge on USP9X levels (Fig 5A). Previously, it was shown that USP9X localizes predominately to the cytoplasm (where it controls the localization/activity of MARCH7[36,37,38]), but its targeting to the centrosome has not been documented. We confirmed that USP9X is indeed mostly cytoplasmic; nevertheless, this protein is also detected at the centrosome where it co-localizes with a centriolar marker centrin (Figs 5B and 6C). Depletion of NPHP5 specifically disrupted the centrosomal localization of USP9X (Fig 5B), suggesting that a pool of cytoplasmic USP9X is recruited to the centrosome where it might serve to deubiquitinate NPHP5.

### Impaired binding to USP9X leads to K63 ubiquitination of NPHP5

To better understand the role and requirements of USP9X in regulating NPHP5 ubiquitination, we examined the protein levels and localization of NPHP5 and USP9X, along with the interaction between the two proteins, across the cell cycle. Although NPHP5 becomes K48 and K63 ubiquitinated when USP9X is lost or inhibited, we found that endogenous NPHP5 levels remain relatively constant throughout the cell cycle (Fig 6A and 6B). Neither cycloheximide nor MG132 dramatically destabilized or stabilized NPHP5 (control lanes; Fig 4C and 4D), suggesting that this protein is highly stable and has a low turnover rate when USP9X is not perturbed. In terms of localization, NPHP5 was highly enriched at the centrosome during interphase (S6 Fig). Its centrosomal staining became fuzzy in late G2 and disappeared in mitosis (S6 Fig), in agreement with our previous study[25]. Thus, NPHP5 is delocalized but is not down-regulated in mitosis. For USP9X, we found that while its levels remain the same throughout the cell cycle (Fig 6A and 6B), the protein localizes to the centrosome in G0, G1 and S only (Fig 6C). The absence of centrosomal USP9X staining in G2 and M (Fig 6C) suggests that it is lost from the organelle at a much earlier point in the cell cycle than NPHP5. Consistent with this, the interaction between NPHP5 and USP9X was strong in G0, G1 and S and became substantially weaker in G2 and M (Fig 6A and 6B). Collectively, our data support the idea that USP9X associates with NPHP5 at the centrosome for most of the cell cycle. This enzyme then dissociates from the centrosome in the G2 and M phase, concomitant with its decreased affinity for NPHP5. During mitosis, NPHP5 also becomes delocalized.

Because USP9X deubiquitinates NPHP5 (Fig 3), we hypothesize that NPHP5 is prone to ubiquitination when its association with USP9X is compromised in G2/M. The levels of NPHP5 ubiquitination were indeed elevated in mitosis (Fig 6D), and this increase was specifically attributed to the attachment of K63-Ub, but not K48-Ub chains, to NPHP5 (Fig 6E). Thus, our data suggest that K63 ubiquitination of NPHP5 is likely responsible for its delocalization from the centrosome in the mitotic phase of the cell cycle.

To further prove that NPHP5 is prone to K63 ubiquitination when it is not binding to USP9X, we conducted a serious of experiments with 20–598, a NPHP5 mutant refractory to USP9X binding (Fig 2C and 2D). Curiously, 20–598 was more highly ubiquitinated compared to wild type NPHP5 (S7A Fig) and underwent predominantly K63 ubiquitination (S7A Fig). Despite possessing an intact centrosome localization domain[25] and expressing at levels similar to wild type NPHP5, 20–598 was poorly targeted to the centrosome (S7B and S7C Fig). Weak centrosomal staining of the mutant protein was seen in ~35% of transfected cells, while no centrosomal staining was observed in the remaining ~65%.

### MARCH7 induces K48 ubiquitination of NPHP5

**Fig 7:**
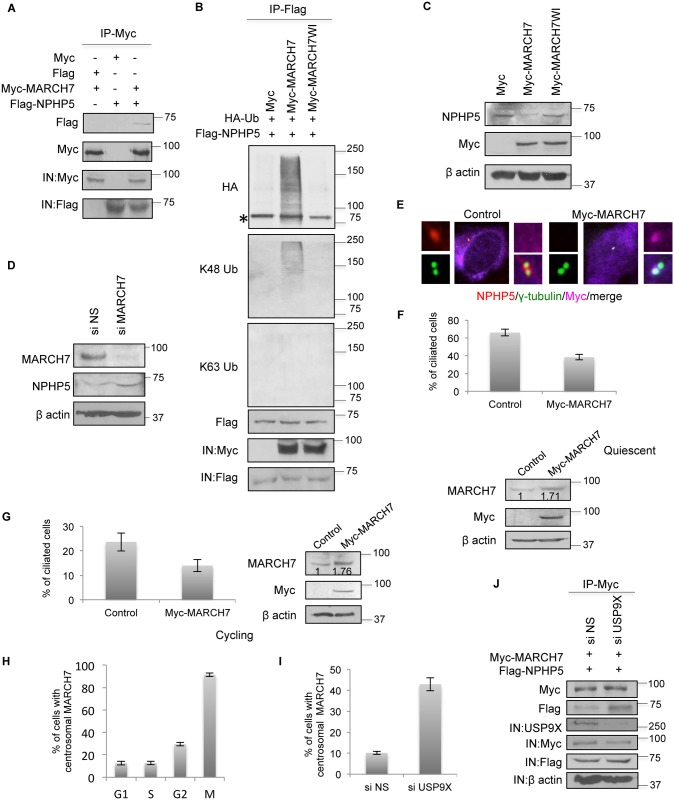
NPHP5 is K48 ubiquitinated by MARCH7. **A)** Flag or Flag-NPHP5 was co-expressed with Myc or Myc-MARCH7 in HEK293 cells. Lysates were immunoprecipitated with anti-Myc antibody and Western blotted with the indicated antibodies. IN, input. **B)** Myc, Myc-MARCH7 wild type or catalytically inactive mutant (WI) was co-expressed with Flag-NPHP5 and HA-Ub in HEK293 cells. Lysates were immunoprecipitated with anti-Flag antibody in 1% SDS and Western blotted with the indicated antibodies. IN, input. **C)** HEK293 cells were transfected with Myc, Myc-MARCH7 wild type or mutant. Lysates were Western blotted with the indicated antibodies. β actin was used as a loading control. **D)** HEK293 cells were transfected with control (NS) or MARCH7 siRNA. Lysates were Western blotted with the indicated antibodies. β actin was used as a loading control. **E)** Cycling RPE-1 cells expressing an irrelevant Myc tagged protein (control) or Myc-MARCH7 were stained with Myc (violet), NPHP5 (red) and γ-tubulin (green). **F-G)** An irrelevant Myc tagged protein (control) or Myc-MARCH7 was expressed in quiescent **F)** or cycling **G)** RPE-1 cells. (**F**, top; **G**, left) Cells were stained with Myc and glutamylated tubulin (GT335) and the percentage of Myc positive cells with cilia was determined. (**F**, bottom; **G** right) Lysates were Western blotted with the indicated antibodies. β actin was used as a loading control. Numbers indicate total MARCH7 levels relative to control. **H)** The percentage of RPE-1 cells with centrosomal MARCH7 staining across the cell cycle is presented. **I)** The percentage of cycling RPE-1 cells with centrosomal MARCH7 staining after transfection with control (NS) or USP9X siRNA is presented. **J)** HEK293 cells were transfected with control (NS) or USP9X siRNA and plasmids expressing Myc-MARCH7 and Flag-NPHP5. Lysates were immunoprecipitated with anti-Myc antibody and Western blotted with the indicated antibodies. IN, input. In **F-I)** at least 100 cells were counted per condition, and error bars represent average of three independent experiments. Asterisks indicate non-specific bands.

We have thus far demonstrated that NPHP5 becomes ubiquitinated when it is not protected by USP9X: depletion/inhibition of USP9X triggers K48/K63 ubiquitination of NPHP5 and protein degradation/mis-localization, whereas impaired binding to USP9X provokes K63 ubiquitination and protein mis-localization. To reconcile these observations and to identify enzymes responsible for the differential ubiquitination of NPHP5, we examined the connection between NPHP5 and the two E3 ligases, MARCH7 and BBS11 (Fig 1A). We first validated the interaction between NPHP5 and MARCH7 (Fig 7A). This interaction is likely direct since the two proteins bound to each other *in vitro* (S8A Fig). Unlike the catalytically inactive mutant[32], wild type MARCH7 was able to trigger NPHP5 ubiquitination (Fig 7B). More specifically, MARCH7 induced K48 but not K63 ubiquitination of NPHP5 (Fig 7B), resulting in decreased protein levels (Fig 7C). Conversely, depletion of MARCH7 elevated NPHP5 protein levels (Fig 7D). Enforced expression of MARCH7 reduced the fluorescence intensity of NPHP5 at the centrosome (Fig 7E) and suppressed cilia formation in cycling and quiescent RPE-1 cells (Fig 7F and 7G). Although MARCH7 is predominately nuclear and cytoplasmic[32,39], a weak endogenous signal could be detected at the centrosome in a small percentage of interphase cells (Fig 7H and S8B Fig). This minute amount of MARCH7 was not sufficient to induce substantial K48 ubiquitination of NPHP5 (Fig 6E). During mitosis, a strong centrosomal MARCH7 signal could be seen in most cells (Fig 7H and S8B Fig). Remarkably, we found that depletion of USP9X leads to an accumulation of MARCH7 at the centrosome in the majority of cells irrespective of the cell cycle (Fig 7I and S8C Fig). The interaction between MARCH7 and NPHP5 became stronger upon USP9X depletion (Fig 7J). Our results suggest that MARCH7 has subtle effects on NPHP5 under normal, unperturbed conditions. Upon USP9X depletion or inhibition, however, NPHP5 is exposed to E3 ligases. MARCH7 becomes mis-targeted to the centrosome where it adds K48-Ub chains to NPHP5 (Figs 1F, 1G and 3D). Meanwhile, another enzyme might be responsible for putting K63-Ub chains onto NPHP5 (Figs 1F, 1G and 3D).

### BBS11 induces K63 ubiquitination of NPHP5

**Fig 8:**
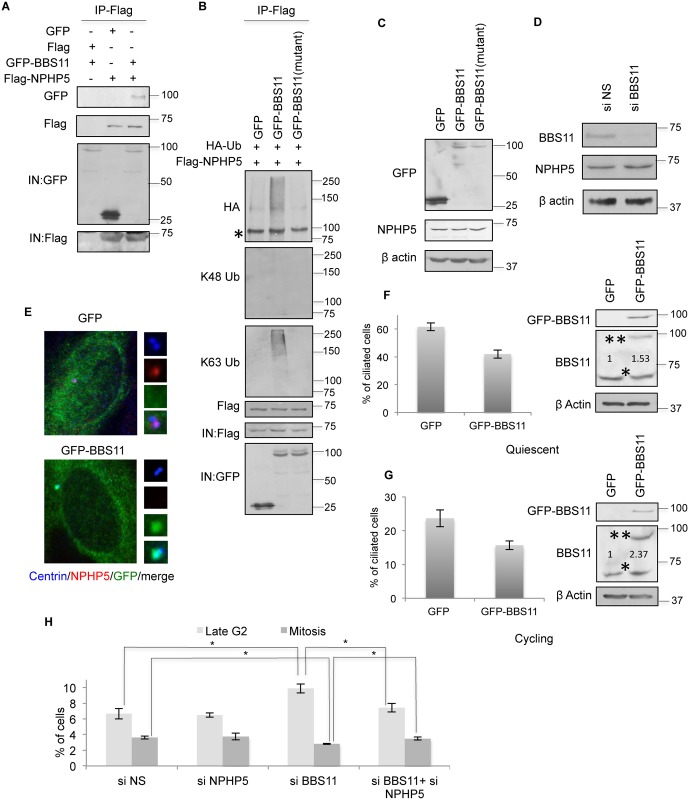
NPHP5 is K63 ubiquitinated by BBS11. **A)** Flag or Flag-NPHP5 was co-expressed with GFP or GFP-BBS11 in HEK293 cells. Lysates were immunoprecipitated with anti-Flag antibody and Western blotted with the indicated antibodies. IN, input. **B)** GFP, GFP-BBS11 wild type or catalytically inactive mutant (C20AC39AH41A; mutant) was co-expressed with Flag-NPHP5 and HA-Ub in HEK293 cells. Lysates were immunoprecipitated with anti-Flag antibody in 1% SDS and Western blotted with the indicated antibodies. IN, input. Asterisk indicates a non-specific band. **C)** HEK293 cells were transfected with GFP, GFP-BBS11 wild type or mutant. Lysates were Western blotted with the indicated antibodies. β actin was used as a loading control. **D)** HEK293 cells were transfected with control (NS) or BBS11 siRNA. Lysates were Western blotted with the indicated antibodies. β actin was used as a loading control. **E)** Cycling RPE-1 cells expressing GFP or GFP-BBS11 were stained with antibodies against GFP (green), NPHP5 (red) and centrin (blue). **F-G)** GFP or GFP-BBS11 was expressed in quiescent **F)** or cycling **G)** RPE-1 cells. (Left) Cells were stained with GFP and glutamylated tubulin (GT335) and the percentage of GFP positive cells with cilia was determined. (Right) Lysates were Western blotted with the indicated antibodies. β actin was used as a loading control. Numbers indicate total BBS11 levels relative to control. * and ** indicate endogenous and recombinant BBS11, respectively. **H)** The percentage of control (NS), NPHP5, BBS11 or BBS11 and NPHP5 siRNA-treated RPE-1 cells in late G2 or M phase was determined. * indicates p<0.01. In **F-H)** at least 100 cells were counted per condition, and error bars represent average of three independent experiments.

Next, we validated the interaction between NPHP5 and BBS11 (Fig 8A), which was reported to be weak [40], and showed that these proteins directly interact *in vitro* (S9A Fig). Wild type but not catalytically inactive mutant BBS11[41] induced NPHP5 ubiquitination (Fig 8B). In striking contrast to MARCH7, BBS11 specifically provoked K63 rather than K48 ubiquitination of NPHP5 (Fig 8B). Endogenous BBS11 localized to the centrosome in all cell cycle phases examined (S9B Fig), suggesting that it might have the capacity to ubiquitinate NPHP5 throughout the cell cycle. Consistent with the notion that BBS11 K63-ubiquitinates NPHP5, neither ectopic expression nor depletion of BBS11 impinged on the protein levels of NPHP5 (Fig 8C and 8D). Ectopic expression of BBS11 triggered the delocalization of NPHP5 (Fig 8E) and suppressed cilia formation in cycling and quiescent cells (Fig 8F and 8G). Depletion of BBS11 enhanced the localization of the 20–598 mutant of NPHP5 to the centrosome (S7D Fig) and, unlike depletion of MARCH7, caused a dramatic accumulation of centrosomal NPHP5 in late G2 cells (S10 Fig). Furthermore, depletion of BBS11 induced late G2 arrest/delay (Fig 8H), in agreement with results reported earlier[42], and this phenotype could be reversed by co-depletion of NPHP5 (Fig 8H). In a subset of BBS11-depleted cells that have managed to slip into mitosis, persistent centrosomal NPHP5 staining could be observed (S9C Fig). Taken together, our data suggest that BBS11 catalyzes the addition of K63-Ub chains to NPHP5, leading to its delocalization from the centrosome without affecting protein levels. Furthermore, timely delocalization of NPHP5 appears to be crucial for proper mitotic entry.

### Antagonistic effects of USP9X and MARCH7/BBS11 on NPHP5

On the basis of our findings that NPHP5 is deubiquitinated by USP9X, K48-ubiquitinated by MARCH7 and K63-ubiquitinated by BBS11, we asked whether simultaneous ablation of MARCH7 and/or BBS11 might override the effects of USP9X loss on NPHP5. Depletion of USP9X induced down-regulation and mis-localization of NPHP5 (Fig 4A–4D and S4, S5A, S5E, S5F, S11A and S11B Figs). Co-depletion of MARCH7 reinstated protein levels but not the centrosomal localization of NPHP5 (S11A and S11B Fig), whereas co-depletion of BBS11 could barely restore localization, presumably because protein levels remained low (S11A and S11B Fig). Co-depletion of MARCH7 and BBS11, however, drastically restored the centrosomal localization and protein levels of NPHP5 (S11A and S11B Fig). We have thus established a functional connection between USP9X, MARCH7, BBS11 and NPHP5.

## Discussion

**Fig 9:**
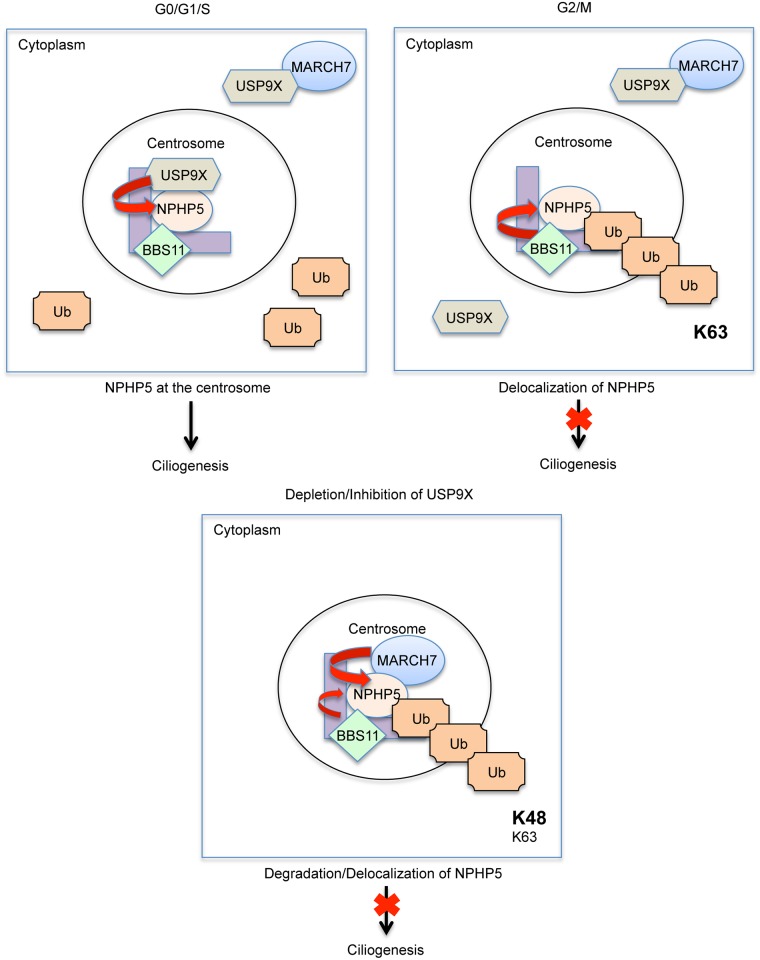
Model depicting the role of ubiquitination and deubiquitination in controlling NPHP5-mediated ciliogenesis. (Top left) In the G0/G1/S phase, a pool of cytoplasmic USP9X is recruited to the centrosome by NPHP5. MARCH7 is held in the cytoplasm by USP9X, whereas BBS11 is localized to the centrosome. Because centrosomal USP9X actively deubiquitinates NPHP5, NPHP5 is stabilized and cilia assembly is favoured. (Top right) In the G2/M phase, USP9X dissociation from the centrosome makes NPHP5 prone to ubiquitination. NPHP5 undergoes K63-ubiquitination by BBS11 and becomes delocalized, and cilia disassembly is favoured. MARCH7 is sequestered by USP9X in the cytoplasm at G2. MARCH7 goes to the centrosome in mitosis, but NPHP5 is already delocalized. (Bottom) When USP9X is depleted or inhibited, NPHP5 is prone to ubiquitination. MARCH7 becomes aberrantly translocated to the centrosome wherein it K48-ubiquitinates NPHP5. BBS11 can K63-ubiquitinate NPHP5 at the same time. As a result, NPHP5 is degraded and delocalized, and cilia disassembly is favoured.

We present here the spatial and temporal regulation of NPHP5. We show that there is a significant correlation between the sub-cellular localization of NPHP5, the biological activity of NPHP5, and the capacity of cells to possess cilia. Our data suggest that at G0/G1/S when cilia are present, NPHP5 directly recruits a fraction of cytoplasmic USP9X to the centrosome which in turn protects NPHP5 from ubiquitination (Fig 9). In the G2/M phase when cilia formation is not favourable, USP9X dissociates from the centrosome, making NPHP5 vulnerable to ubiquitination. We identify two E3 ligases, MARCH7 and BBS11 that exhibit distinct localization patterns and exert different effects on NPHP5. In contrast to MARCH7 which is mostly a nuclear/cytoplasmic protein, BBS11 is present at the centrosome throughout the cell cycle. We believe that BBS11 is the enzyme responsible for K63-ubiquitinating NPHP5 and triggering its delocalization at late G2/M (Fig 9). The molecular cause of USP9X dissociation from NPHP5 is currently under investigation.

Why is it necessary for NPHP5 to move out of the centrosome in mitosis? NPHP5 is known to be a positive regulator of ciliogenesis[24,25]. Given that the presence of cilia is incompatible with spindle formation and/or function[43], failure to delocalize NPHP5 might interfere with the removal of downstream ciliogenesis events or cilia disassembly and affect cell cycle progression. In support of this idea, our studies show that a loss of BBS11 causes an accumulation of NPHP5 at the centrosome at late G2, which in turn compromises mitotic entry. We hereby propose that the delocalization of NPHP5 triggered by BBS11 represents a novel mechanism that acts in concert with the recently characterized Nek-Kif24 pathway[44] to favour cilia disassembly at G2/M.

The localization/activity of MARCH7 is controlled by two different DUBs, USP7 in the nucleus and USP9X in the cytoplasm[32]. In particular, it is known that USP9X, which is predominantly cytoplasmic, interacts with and sequesters MARCH7 in this compartment (Fig 9 and [32]). Although we demonstrate that NPHP5 is a substrate of MARCH7, these two proteins are mostly found in distinct sub-cellular compartments. Indeed, MARCH7 is barely detectable at the centrosome during interphase. MARCH7 does become highly enriched at the centrosome during mitosis, but NPHP5 becomes K63 ubiquitinated by BBS11 and is delocalized at this juncture. Thus, under normal and unperturbed conditions, MARCH7 does not K48-ubiquitinate NPHP5 to a significant degree (Fig 9), which could explain the observed stability of NPHP5. A priori, MARCH7 could contribute to the low turnover of NPHP5 by targeting a small fraction of this protein for degradation. When USP9X is depleted or inhibited, however, the localization/activity of MARCH7 is dramatically altered. MARCH7 becomes aberrantly translocated to the centrosome where both MARCH7 and BBS11 can now ubiquitinate NPHP5 (Fig 9). MARCH7 induces K48 ubiquitination of NPHP5 and protein degradation, while BBS11 triggers K63 ubiquitination and protein delocalization.

Previously, it has been reported that USP9X localizes to cilia and that depletion of USP9X does not impair ciliogenesis in human fibroblasts[45]. At first glance, these observations appear to contradict our findings. We speculate that these discrepancies could be attributed to the knockdown efficiency and the use of different cell lines (fibroblasts versus RPE-1 cells; see [32]) which give different USP9X sub-cellular localization patterns. In our hands, depletion of USP9X in RPE-1 cells was extremely robust.

In summary, the ubiquitination status and hence the stability, localization and biological activity of NPHP5 is controlled by two E3 ligases (MARCH7 and BBS11) and a DUB (USP9X). These studies raise the intriguing possibility that targeting certain DUBs or E3 ligases might represent a novel strategy to manipulate cilia assembly pathways and treat cilia-related diseases.

## Materials and methods

### Cell culture and plasmids

HEK293, U2OS and hTERT RPE-1 cells were grown in DMEM supplemented with 10% FBS at 37°C in a humidified 5% CO_2_ atmosphere. Plasmids expressing GFP-NPHP5, Flag-NPHP5 and Flag-NPHP5 truncates were described previously[25]. Mouse USP9X (S. Wood), mouse USP9X C1566S (S. Wood) and human BBS11 cDNAs were sub-cloned into mammalian vector pEGFP-C1 to generate pEGFP-C1-USP9X, pEGFP-C1-USP9X(C1566S) and pEGFP-C1-BBS11 respectively. Mutations in BBS11 (C20AC39AH41A) and NPHP5 (K19RK31RK33RK205RK263RK528R and 20–598) were introduced into full-length cDNAs by PCR mutagenesis and sub-cloned into pEGFP-C1, pEGFP-N1 or pCBF-Flag. All constructs were verified by DNA sequencing. Plasmids expressing the following proteins were obtained or purchased: HA-Ub (J. Archambault); Flag-Ub, Flag-Ub KallR, Flag-Ub K48R, Flag-Ub K63R and Flag-Ub K48RK63R (Division of Signal Transduction Therapy, University of Dundee); Myc-MARCH7 and Myc-MARCH7WI (P. Lehner).

### Antibodies

Antibodies used in this study included anti-HA, anti-NPHP5, anti-Myc, anti-MARCH7, anti-γ tubulin-FITC (Santa Cruz); anti-Flag, anti-GFP, anti-β actin, (Sigma); anti-CP110, anti-USP9X, anti-CEP290 (Bethyl Laboratories); anti-BBS11 (Thermo Fisher), anti-centrin, anti-K48 Ub (Millipore); anti-K63 Ub (Abcam); anti-Ub (Dako); anti-IFT88 (Proteintech); anti-glutamylated tubulin GT335, anti-acetylated tubulin and anti-Ki67 (Invitrogen). Two additional anti-NPHP5 antibodies (IRCM1 and IRCM2) were also used[25].

### Mass spectrometric identification of NPHP5-associated proteins, ubiquitination sites and Ub chain linkages

To identify NPHP5-interacting proteins, HEK293 cells expressing Flag-NPHP5 were lysed in a buffer containing 50 mM HEPES/pH 7.4, 250 mM NaCl, 5 mM EDTA/pH 8, 0.1% NP-40, 1 mM DTT, 0.2 mM AEBSF, 2 μg/ml leupeptin, 2 μg aprotinin, 10 mM NaF, 50 mM β-glycerophosphate and 10% glycerol for 30 minutes at 4°C. Lysates were immunoprecipitated with anti-Flag agarose beads (Sigma) for 2 hours at 4°C. Bounded proteins were eluted with Flag peptide for 30 minutes, and the resultant eluates were precipitated with trichloroacetic acid and fractionated by sodium dodecyl sulfate–polyacrylamide gel electrophoresis. Gel slices containing polypeptides were excised after Coomassie staining and subjected to proteolytic digestion mass spectrometric analysis. To identify ubiquitination sites on, and Ub chains linked to, NPHP5, the procedure was similar except that 5 mM NEM (Bioshop) was added to the lysis buffer. After immunoprecipitation, beads were washed three times with 500 mM NaCl to disrupt interacting proteins from co-immunoprecipitating with Flag-NPHP5. Gel slices containing polypeptides ≥75 kDa were excised for mass spectrometric analysis. Analyses were performed at the IRCM mass spectrometry core facility by micro-capillary liquid chromatography-mass spectrometry/mass spectrometry. LC-MS peptide quantitation was performed by manual integration of the extracted ion chromatogram of each peptide using Qual Browser/Xcalibur version 2.2 (ThermoFisher Scientific, Waltham, MA, USA).

### Immunoprecipitation, immunoblotting and immunofluorescence microscopy

Immunoprecipitation, immunoblotting and immunofluorescence were performed as described previously[40,46].

### Cell cycle synchronization and fluorescence-activated cell sorting analysis

To obtain cells synchronized in G1, S, G2 and M phases, cells were treated with 0.4 mM mimosine for 24 hours, 2 mM HU for 24 hours and released for 4 hours, 2 mM HU for 24 hours and released for 9 hours or 15 μM RO 3306 for 24 hours, and 40 ng/ml nocodazole for 24 hours, respectively. For RPE-1, cells grown in the presence of serum (cycling) or brought to quiescence by serum starvation for 48–72 hours (quiescent) were stained with Ki67, a cellular marker for proliferation. A high percentage of Ki67-positive cells is indicative of cell proliferation, whereas a low percentage is indicative of cell cycle exit. Cell cycle distribution was performed by fluorescence-activated cell sorting as described previously[25]. To prevent proteasomal degradation and protein synthesis, cells were treated with 10 μM MG132 and 10 mg/ml cycloheximide, respectively, for a maximum of 8 hours.

### RNA interference

Transfection of siRNA was performed using siImporter (Millipore) per manufacturer's instructions. Target siRNA sequences were: non-specific (NS) control: 5′-AATTCTCCGAACGTGTCACGT-3′; USP9X-2: 5′-ACACGATGCTTTAGAATTT-3′; USP9X-3: 5′-GTACGACGATGTATTCTCA-3′; NPHP5: 5′-ACCCAAGGATCTTATCTAT-3′. USP9X-2 oligo was used unless stated otherwise. ON-TARGETplus SMARTpool for MARCH7 and BBS11 were used. All siRNAs were purchased from Dharmacon.

### Reverse transcriptase-polymerase chain reaction (RT-PCR)

Total RNA was prepared from HEK293 cell culture using Trizol reagent (Invitrogen). Messenger RNA was reverse-transcribed to cDNA (42°C for 1 hour, 50°C for 1 hour and 90°C for 10 minutes) using random hexamers and Superscript II reverse transcriptase (Applied Biosystems, Carlsbad, USA). A negative control RT- reaction was carried out to establish that the target RNA was not contaminated with DNA. The cDNA product was used as a template for subsequent PCR amplification. Target sequences for NPHP5 were: 5′-GCTTACACAGATATTAAGC-3′, 3′-TCATCTTTTTCTTCAGCCTTA-5′; and for β actin were: 5′-AGAGCTACGTGCCTGAC-3′, 3′-AGCACTGTGTTGGCGTACAG-5′. β actin was used as a control.

### Production of purified proteins

Flag-NPHP5 or GFP-USP9X expressed in HEK293 cells was immunoprecipitated with anti-Flag or anti-GFP antibody. To obtain poly (HA)-ubiquitinated NPHP5, Flag-NPHP5 co-expressed with HA-Ub in HEK293 cells was immunoprecipitated with anti-Flag antibody in the presence of 5 mM NEM to increase the amount of polyubiquitinated products. To obtain BBS11 or MARCH7, endogenous protein was immunoprecipitated from HEK293 cells with anti-BBS11 or anti-MARCH7 antibody. Beads were washed three times with lysis buffer containing 500 mM NaCl to remove interacting proteins from co-immunoprecipitating with the desired protein. For Flag-NPHP5 and poly (HA)-ubiquitinated NPHP5, proteins were eluted with Flag peptide and collected through poly-prep chromatography columns (BioRad).

### *In vivo* ubiquitination assay

*In vivo* ubiquitination assays were performed as described previously[47].

### *In vitro* binding assay

1 μg of purified Flag-NPHP5 was mixed with 1 μg of purified USP9X, BBS11 or MARCH7 bound to beads at 4°C for 2 hours. After extensive washing, bound proteins were analyzed by sodium dodecyl sulfate–polyacrylamide gel electrophoresis and immunoblotting.

### *In vitro* deubiquitination assay

1 μg purified, poly (HA)-ubiquitinated NPHP5 was incubated with 1 μg of purified USP9X bound to beads in 20 μl of reaction buffer (50 mM HEPES-KOH/pH 8, 150 mM KCl, 5% glycerol, 0.01% Triton X-100, and 2 mM DTT) for 2 hours at room temperature. The reaction was terminated by the addition of sodium dodecyl sulfate sample buffer and analyzed by gel electrophoresis and immunoblotting.

### Data and statistical analysis

Graphs were generated in Microsoft Excel. Error bars represent standard error of the mean. For Western blot quantitation, densitometric analyses were performed with ImageJ.

## Supporting information

S1 Fig NPHP5 is polyubiquitinated. **A)** HEK293 cells were transfected with Flag-NPHP5. Lysates were immunoprecipitated with anti-Flag antibody in the absence of presence of 1% SDS. The resultant immunoprecipitates were washed with or without 500 mM NaCl and Western blotted with the indicated antibodies. IN, input. **B)** Flag or Flag-NPHP5 was expressed in HEK293 cells. Lysates treated with or without NEM were immunoprecipitated with anti-Flag antibody in 1% SDS and Western blotted with the indicated antibodies. IN, input.
(TIF)

S2 Fig NPHP5 polyubiquitination is observed in multiple cell lines. **A)** HEK293 cells were transfected with Flag or Flag-NPHP5 and HA or HA-Ub. Lysates were immunoprecipitated with anti-HA antibody in 1% SDS and Western blotted with the indicated antibodies. IN, input. **B-C**) Flag-NPHP5 and HA or HA-Ub were expressed in RPE-1 **B)** or U2OS **C)** cells. Lysates were immunoprecipitated with anti-Flag antibody in 1% SDS and Western blotted with the indicated antibodies. IN, input. Asterisks indicate non-specific bands.
(TIF)

S3 Fig NPHP5 binds to USP9X. GFP, GFP-USP9X or GFP-USP9X mutant (C1566S) was co-expressed with Flag-NPHP5 in HEK293 cells. Lysates were immunoprecipitated with anti-Flag antibody and Western blotted with the indicated antibodies. IN, input.
(TIF)

S4 Fig Depletion of USP9X leads to a loss of centrosomal NPHP5. RPE-1 cells transfected with control (NS) or USP9X siRNA and stained with antibodies against USP9X or NPHP5 (red) and centrin (green). DNA was stained with DAPI (blue). Pictures with multiple cells are shown. Arrows point to centrosomes.
(TIF)

S5 Fig NPHP5 protein but not mRNA levels are regulated by USP9X. **A)** Quantitation of NPHP5 protein levels relative to control (NS) siRNA as in Fig 4B. Average of three independent experiments is shown. **B)** HEK293 cells were transfected with control (NS) or USP9X siRNA and the indicated amount of Flag-NPHP5 constructs in μg. Lysates were Western blotted with the indicated antibodies. β actin was used as a loading control. A decrease in the steady-state levels of Flag-NPHP5 provoked by USP9X depletion can be readily observed when the recombinant protein is expressed at low/moderate levels. **C)** Total RNA isolated from HEK293 cells treated with control (NS) or USP9X siRNA was subjected to reverse transcription followed by PCR. The number of PCR cycles is indicated. β actin was used as a control. RT-, negative control. **D)** Knockdown of USP9X was confirmed by immunoblotting with anti-USP9X antibody. β actin was used as a loading control. **E)** Quantitation of NPHP5 protein levels relative to control (NS) siRNA without MG132 treatment as in Fig 4C. Average of three independent experiments is shown. **F)** Quantitation of NPHP5 protein levels relative to control (NS) siRNA at time 0 as in Fig 4D. Average of three independent experiments is shown. Asterisks indicate p<0.01.
(TIF)

S6 Fig NPHP5 is delocalized from the centrosome in mitosis. RPE-1 cells in different stages of the cell cycle were processed for immunofluorescence with anti-NPHP5 (red) and anti-centrin (green) antibodies. DNA was stained with DAPI (blue).
(TIF)

S7 Fig A NPHP5 mutant refractory to USP9X binding undergoes K63 ubiquitination. **A)** HEK293 cells were transfected with HA-Ub and Flag-NPHP5 wild type or mutant refractory to USP9X binding (20–598). Lysates were immunoprecipitated with anti-Flag antibody in 1% SDS and Western blotted with the indicated antibodies. IN, input. **B)** Cycling RPE-1 cells expressing GFP-NPHP5 wild type or mutant 20–598 were stained with GFP (green) and centrin (blue). **C)** Flag-NPHP5 wild type or mutant 20–598 was expressed in cycling RPE-1 cells. Lysates were Western blotted with the indicated antibodies. β actin was used as a loading control. **D)** Cycling RPE-1 cells transfected with control (NS) or BBS11 siRNA and plasmid expressing GFP-NPHP5 mutant 20–598 were stained with GFP (green), BBS11 (red) and centrin (blue).
(TIF)

S8 Fig MARCH7 binds NPHP5 and is enriched at the centrosome during mitosis or upon USP9X depletion. A) HEK293 cell lysates were immunoprecipitated with anti-IgG (control) or anti-MARCH7 antibody. Endogenous MARCH7 bound to beads was then incubated with purified Flag-NPHP5. Proteins recovered on beads were analyzed by Western blotting with the indicated antibodies. IN, input. **B)** RPE-1 cells in different stages of the cell cycle were processed for immunofluorescence with MARCH7 (green) and CP110 (red) antibodies. DNA was stained with DAPI (blue). **C)** Cycling RPE-1 cells transfected with control (NS) or USP9X siRNA were stained with antibodies against USP9X or CP110 (red) and MARCH7 or centrin (green). DNA was stained with DAPI (blue).
(TIF)

S9 Fig BBS11 binds NPHP5 and is enriched at the centrosome throughout the cell cycle. **A)** HEK293 cell lysates were immunoprecipitated with anti-IgG (control) or anti-BBS11 antibody. Endogenous BBS11 bound to beads was then incubated with purified Flag-NPHP5. Proteins recovered on beads were analyzed by Western blotting with the indicated antibodies. IN, input. **B)** RPE-1 cells in different stages of the cell cycle were processed for immunofluorescence with BBS11 (red) and centrin (green) antibodies. DNA was stained with DAPI (blue). **C)** Cycling RPE-1 cells were transfected with control (NS) or BBS11 siRNA and stained with antibodies against BBS11 or NPHP5 (red) and centrin (green). DNA was stained with DAPI (blue). Prophase cells are shown.
(TIF)

S10 Fig Depletion of BBS11 leads to an accumulation of centrosomal NPHP5 at late G2 phase. Cycling RPE-1 cells were transfected with control (NS), BBS11, MARCH7 or BBS11 and MARCH7 siRNA and stained with antibodies against BBS11, CP110 or NPHP5 (red) and centrin or MARCH7 (green). DNA was stained with DAPI (blue). Late G2 cells are shown.
(TIF)

S11 Fig Antagonistic effects of USP9X and MARCH7/BBS11 on NPHP5. **A)** RPE-1 cells transfected with control (NS), USP9X, or USP9X in combination with MARCH7 and/or BBS11 siRNA, and stained with antibodies against USP9X, NPHP5, BBS11 or CP110 (red) and MARCH7 or centrin (green). DNA was stained with DAPI (blue). **B)** HEK293 cells were transfected with control (NS), USP9X, or USP9X in combination with MARCH7 and/or BBS11 siRNA, Lysates were Western blotted with the indicated antibodies. β actin was used as a loading control.
(TIF)
