# Supplementary figures and images for: USP9X counteracts differential ubiquitination of NPHP5 by MARCH7 and BBS11 to regulate ciliogenesis
Source: PLoS Genet. 2017 May 12;13(5):e1006791. doi: 10.1371/journal.pgen.1006791 (PMC5446187; doi:10.1371/journal.pgen.1006791)

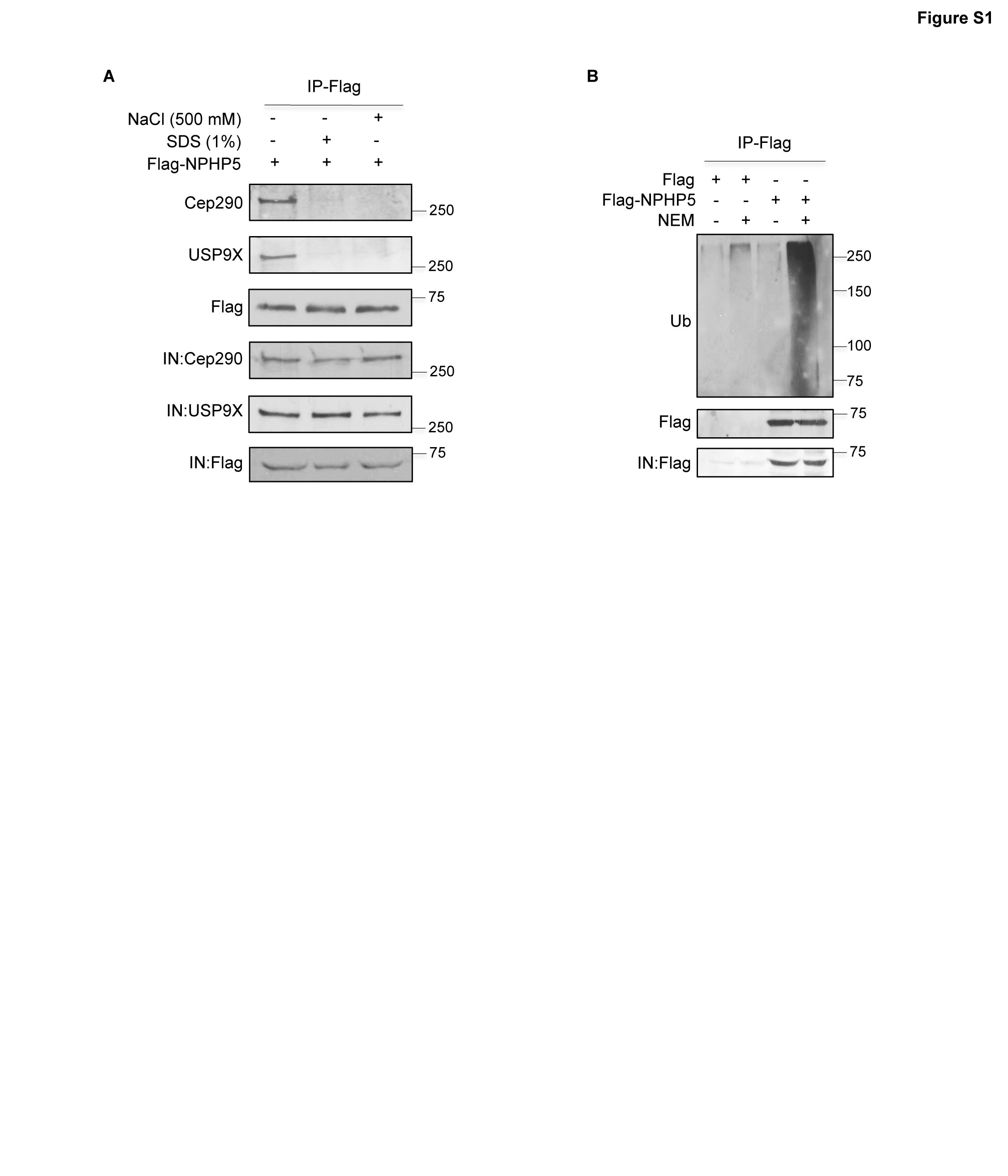

Supplement: S1 Fig — A) HEK293 cells were transfected with Flag-NPHP5. Lysates were immunoprecipitated with anti-Flag antibody in the absence of presence of 1% SDS. The resultant immunoprecipitates were washed with or without 500 mM NaCl and Western blotted with the indicated antibodies. IN, input. B) Flag or Flag-NPHP5 was expressed in HEK293 cells. Lysates treated with or without NEM were immunoprecipitated with anti-Flag antibody in 1% SDS and Western blotted with the indicated antibodies. IN, input. (TIF) [file pgen.1006791.s001.tif]

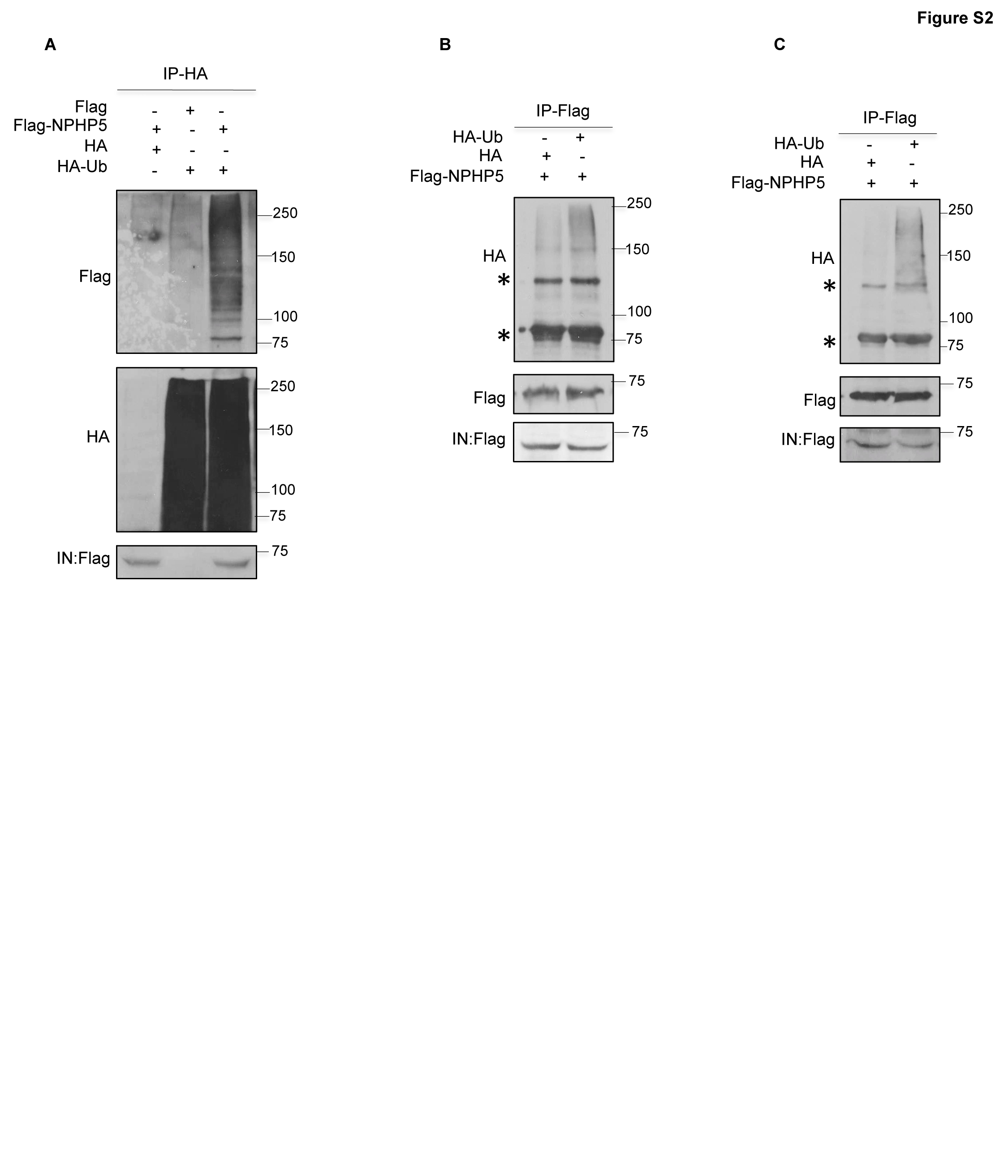

Supplement: S2 Fig — A) HEK293 cells were transfected with Flag or Flag-NPHP5 and HA or HA-Ub. Lysates were immunoprecipitated with anti-HA antibody in 1% SDS and Western blotted with the indicated antibodies. IN, input. B-C) Flag-NPHP5 and HA or HA-Ub were expressed in RPE-1 B) or U2OS C) cells. Lysates were immunoprecipitated with anti-Flag antibody in 1% SDS and Western blotted with the indicated antibodies. IN, input. Asterisks indicate non-specific bands. (TIF) [file pgen.1006791.s002.tif]

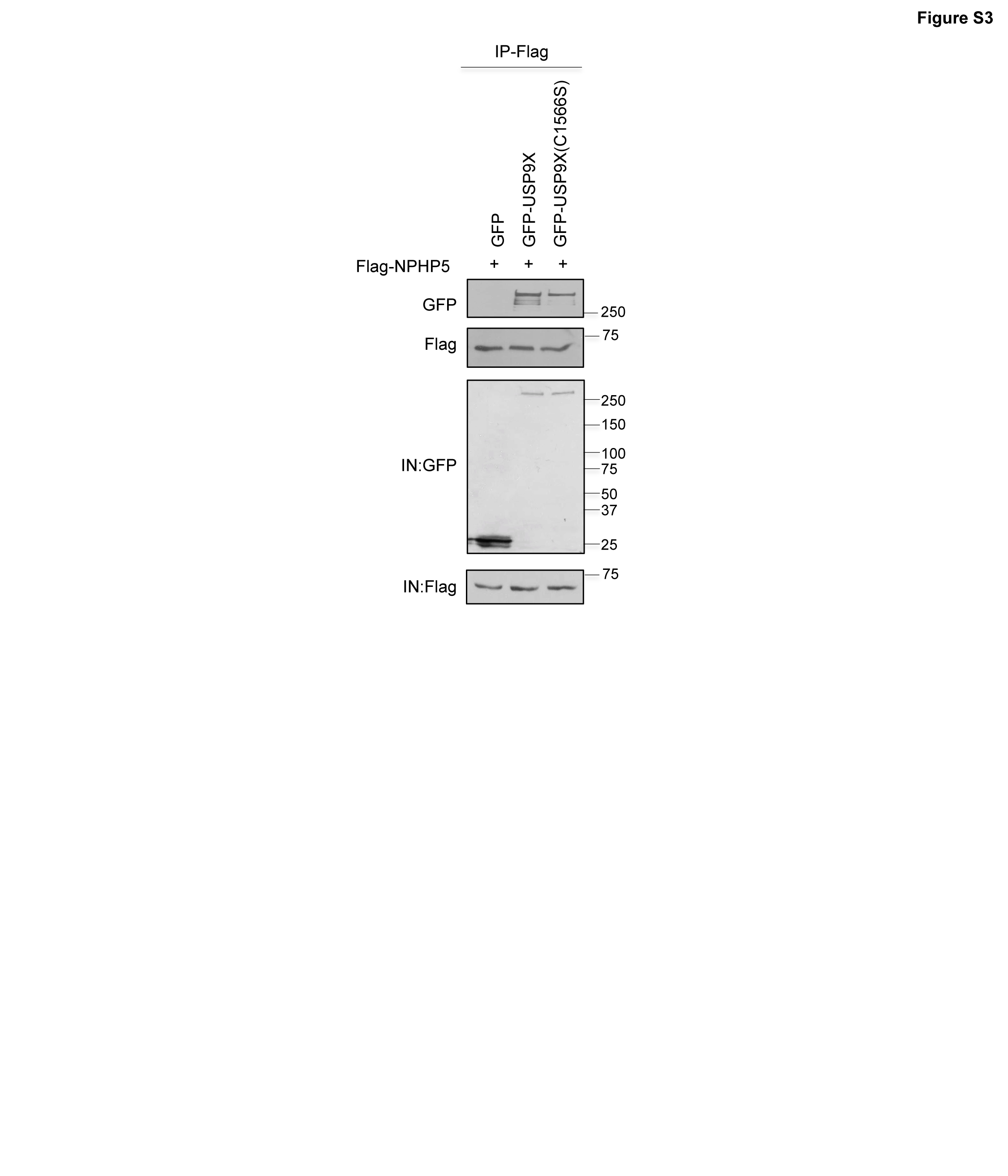

Supplement: S3 Fig — GFP, GFP-USP9X or GFP-USP9X mutant (C1566S) was co-expressed with Flag-NPHP5 in HEK293 cells. Lysates were immunoprecipitated with anti-Flag antibody and Western blotted with the indicated antibodies. IN, input. (TIF) [file pgen.1006791.s003.tif]

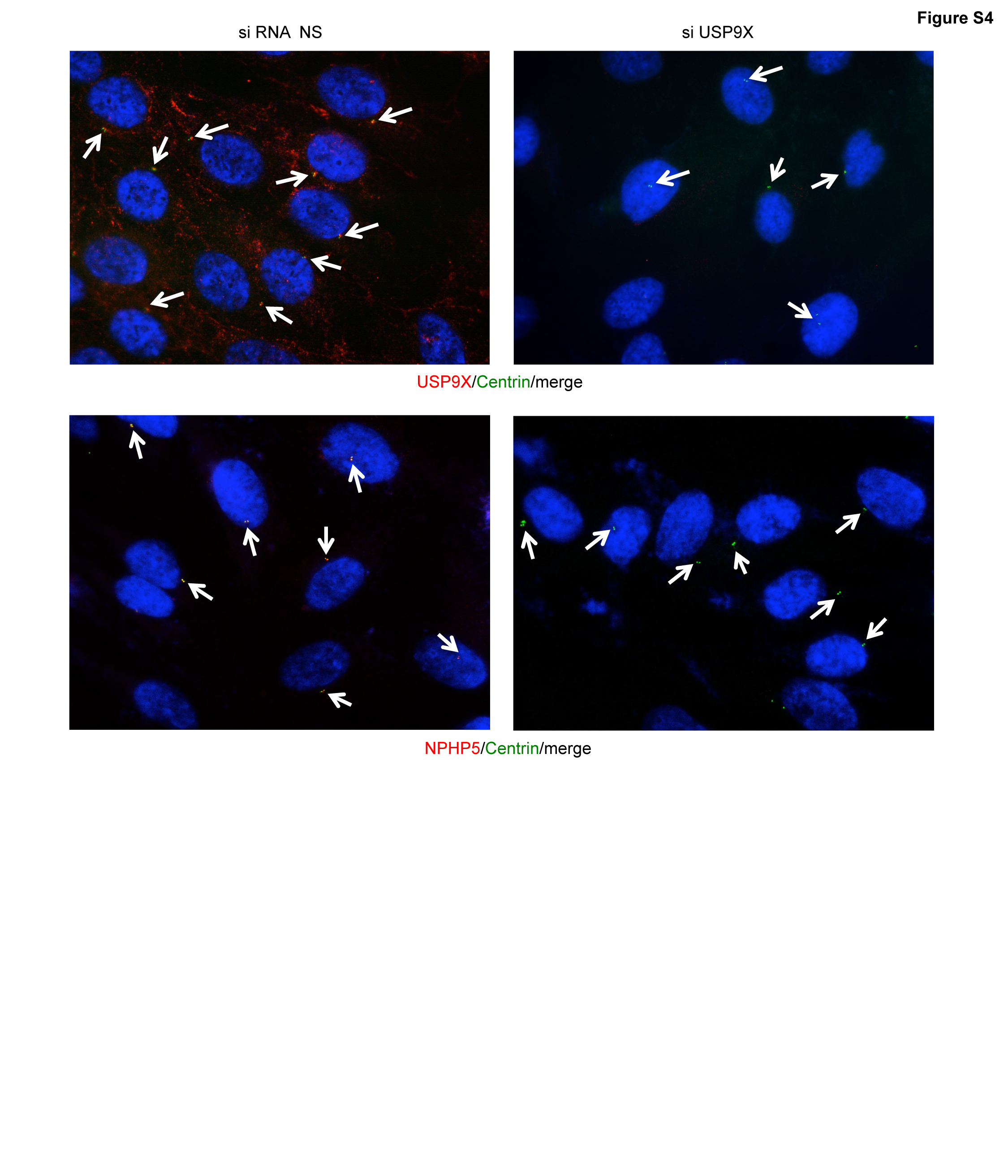

Supplement: S4 Fig — RPE-1 cells transfected with control (NS) or USP9X siRNA and stained with antibodies against USP9X or NPHP5 (red) and centrin (green). DNA was stained with DAPI (blue). Pictures with multiple cells are shown. Arrows point to centrosomes. (TIF) [file pgen.1006791.s004.tif]

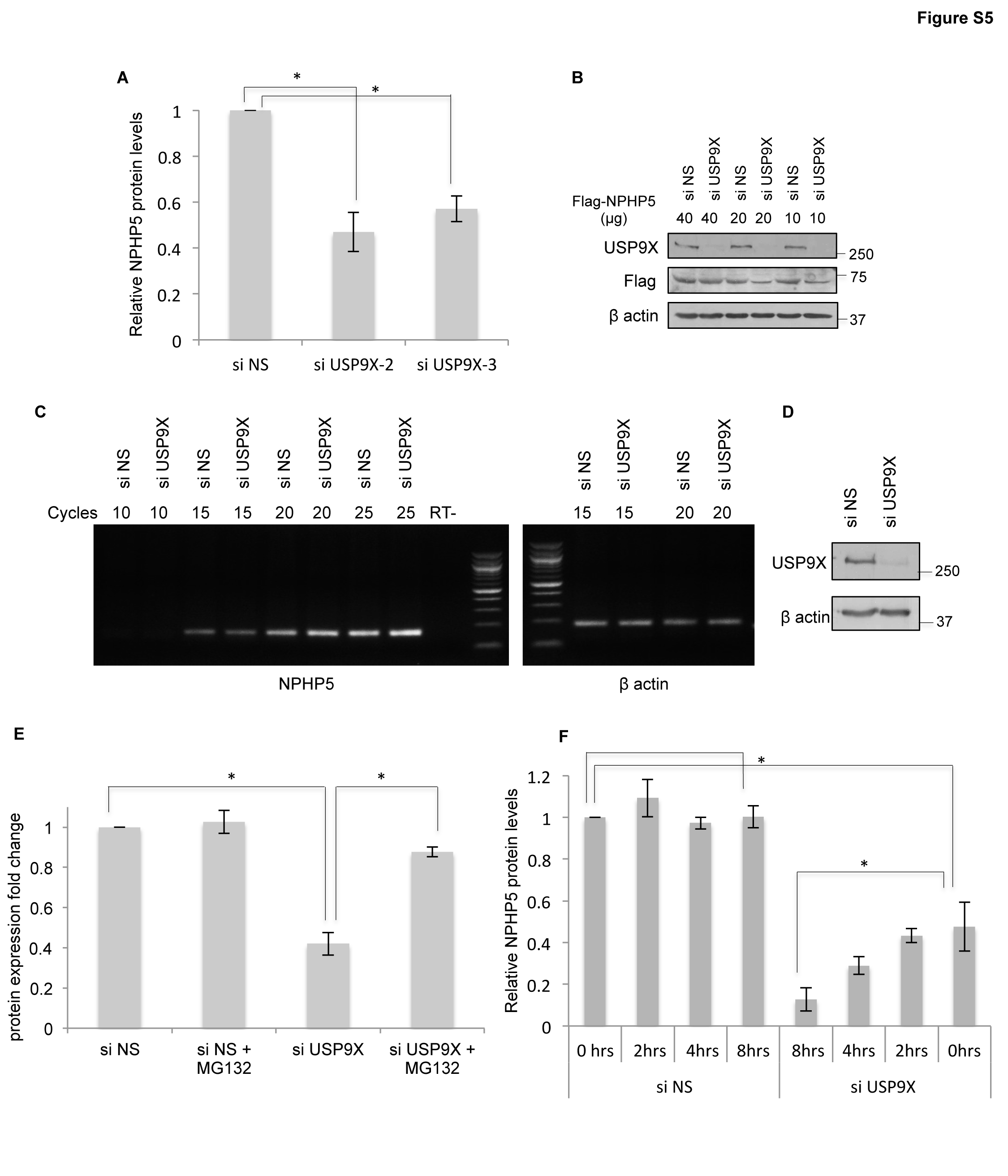

Supplement: S5 Fig — A) Quantitation of NPHP5 protein levels relative to control (NS) siRNA as in Fig 4B. Average of three independent experiments is shown. B) HEK293 cells were transfected with control (NS) or USP9X siRNA and the indicated amount of Flag-NPHP5 constructs in μg. Lysates were Western blotted with the indicated antibodies. β actin was used as a loading control. A decrease in the steady-state levels of Flag-NPHP5 provoked by USP9X depletion can be readily observed when the recombinant protein is expressed at low/moderate levels. C) Total RNA isolated from HEK293 cells treated with control (NS) or USP9X siRNA was subjected to reverse transcription followed by PCR. The number of PCR cycles is indicated. β actin was used as a control. RT-, negative control. D) Knockdown of USP9X was confirmed by immunoblotting with anti-USP9X antibody. β actin was used as a loading control. E) Quantitation of NPHP5 protein levels relative to control (NS) siRNA without MG132 treatment as in Fig 4C. Average of three independent experiments is shown. F) Quantitation of NPHP5 protein levels relative to control (NS) siRNA at time 0 as in Fig 4D. Average of three independent experiments is shown. Asterisks indicate p<0.01. (TIF) [file pgen.1006791.s005.tif]

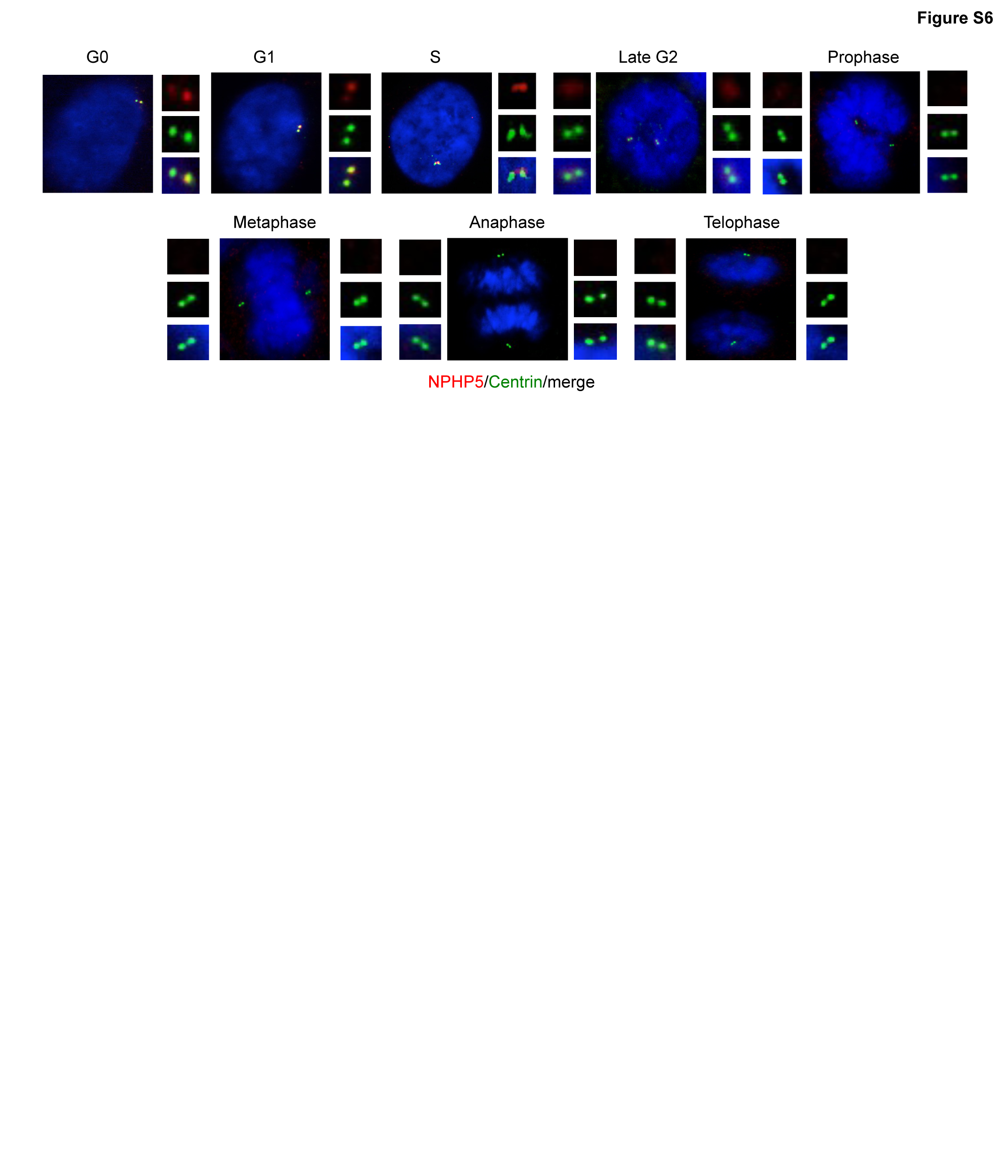

Supplement: S6 Fig — RPE-1 cells in different stages of the cell cycle were processed for immunofluorescence with anti-NPHP5 (red) and anti-centrin (green) antibodies. DNA was stained with DAPI (blue). (TIF) [file pgen.1006791.s006.tif]

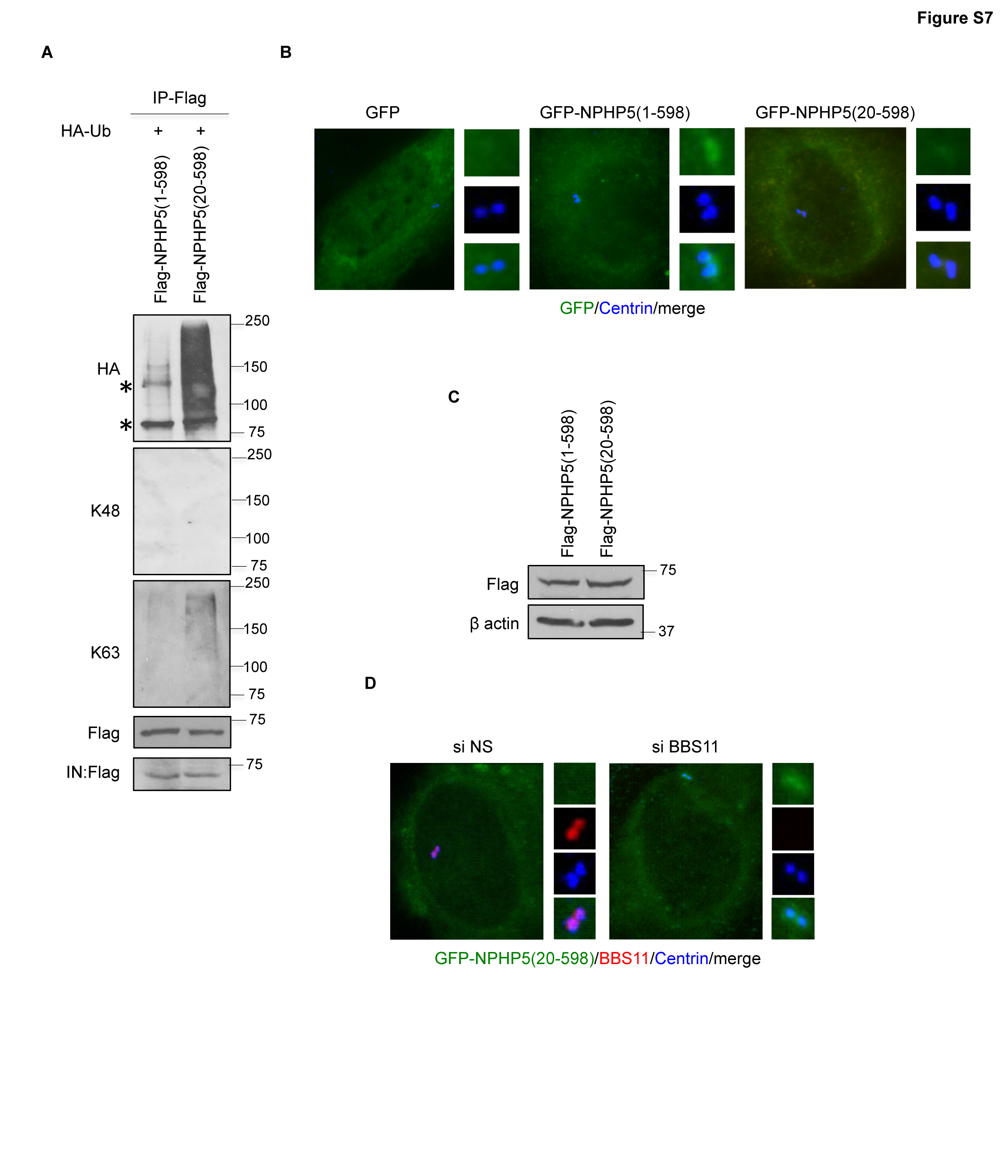

Supplement: S7 Fig — A) HEK293 cells were transfected with HA-Ub and Flag-NPHP5 wild type or mutant refractory to USP9X binding (20–598). Lysates were immunoprecipitated with anti-Flag antibody in 1% SDS and Western blotted with the indicated antibodies. IN, input. B) Cycling RPE-1 cells expressing GFP-NPHP5 wild type or mutant 20–598 were stained with GFP (green) and centrin (blue). C) Flag-NPHP5 wild type or mutant 20–598 was expressed in cycling RPE-1 cells. Lysates were Western blotted with the indicated antibodies. β actin was used as a loading control. D) Cycling RPE-1 cells transfected with control (NS) or BBS11 siRNA and plasmid expressing GFP-NPHP5 mutant 20–598 were stained with GFP (green), BBS11 (red) and centrin (blue). (TIF) [file pgen.1006791.s007.tif]

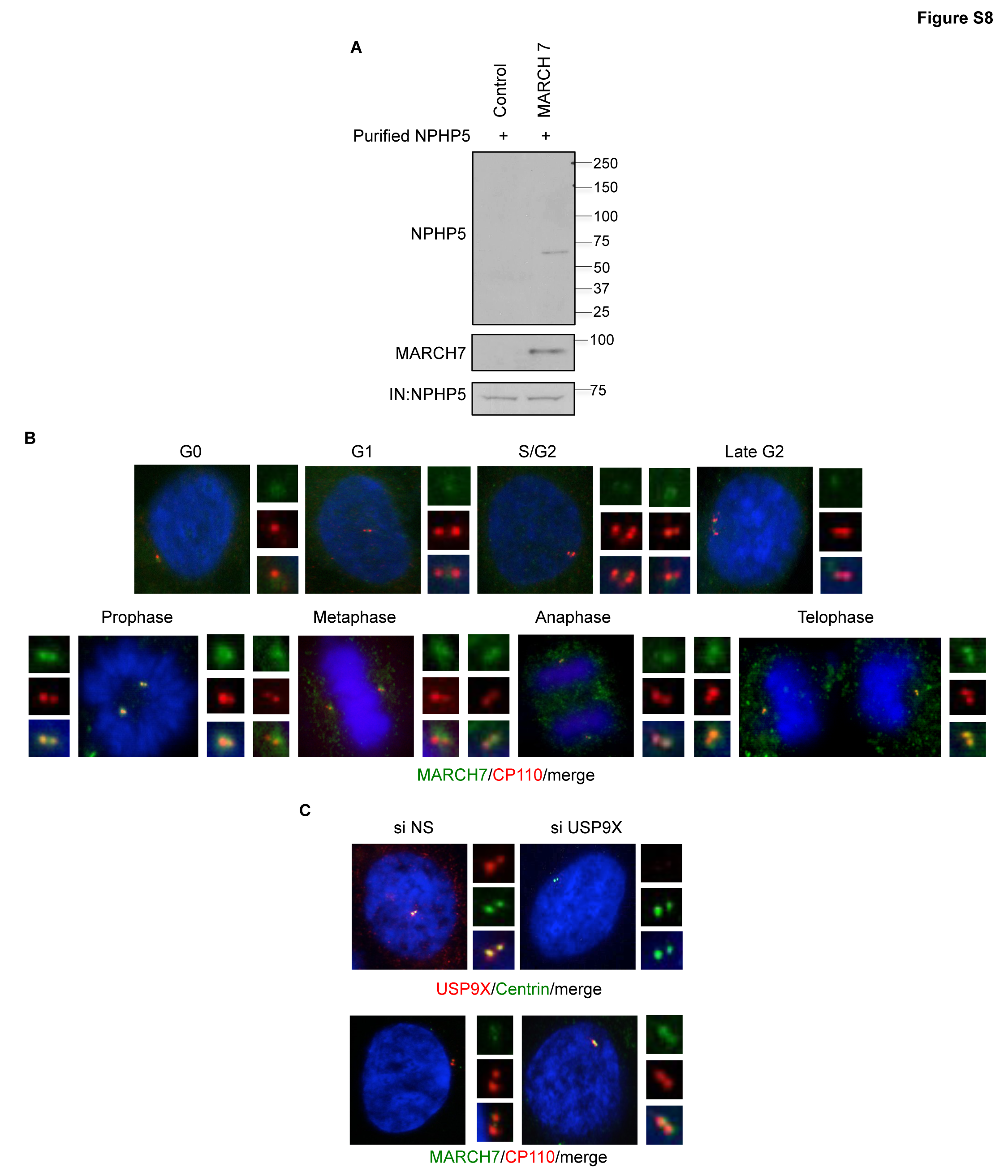

Supplement: S8 Fig — A) HEK293 cell lysates were immunoprecipitated with anti-IgG (control) or anti-MARCH7 antibody. Endogenous MARCH7 bound to beads was then incubated with purified Flag-NPHP5. Proteins recovered on beads were analyzed by Western blotting with the indicated antibodies. IN, input. B) RPE-1 cells in different stages of the cell cycle were processed for immunofluorescence with MARCH7 (green) and CP110 (red) antibodies. DNA was stained with DAPI (blue). C) Cycling RPE-1 cells transfected with control (NS) or USP9X siRNA were stained with antibodies against USP9X or CP110 (red) and MARCH7 or centrin (green). DNA was stained with DAPI (blue). (TIF) [file pgen.1006791.s008.tif]

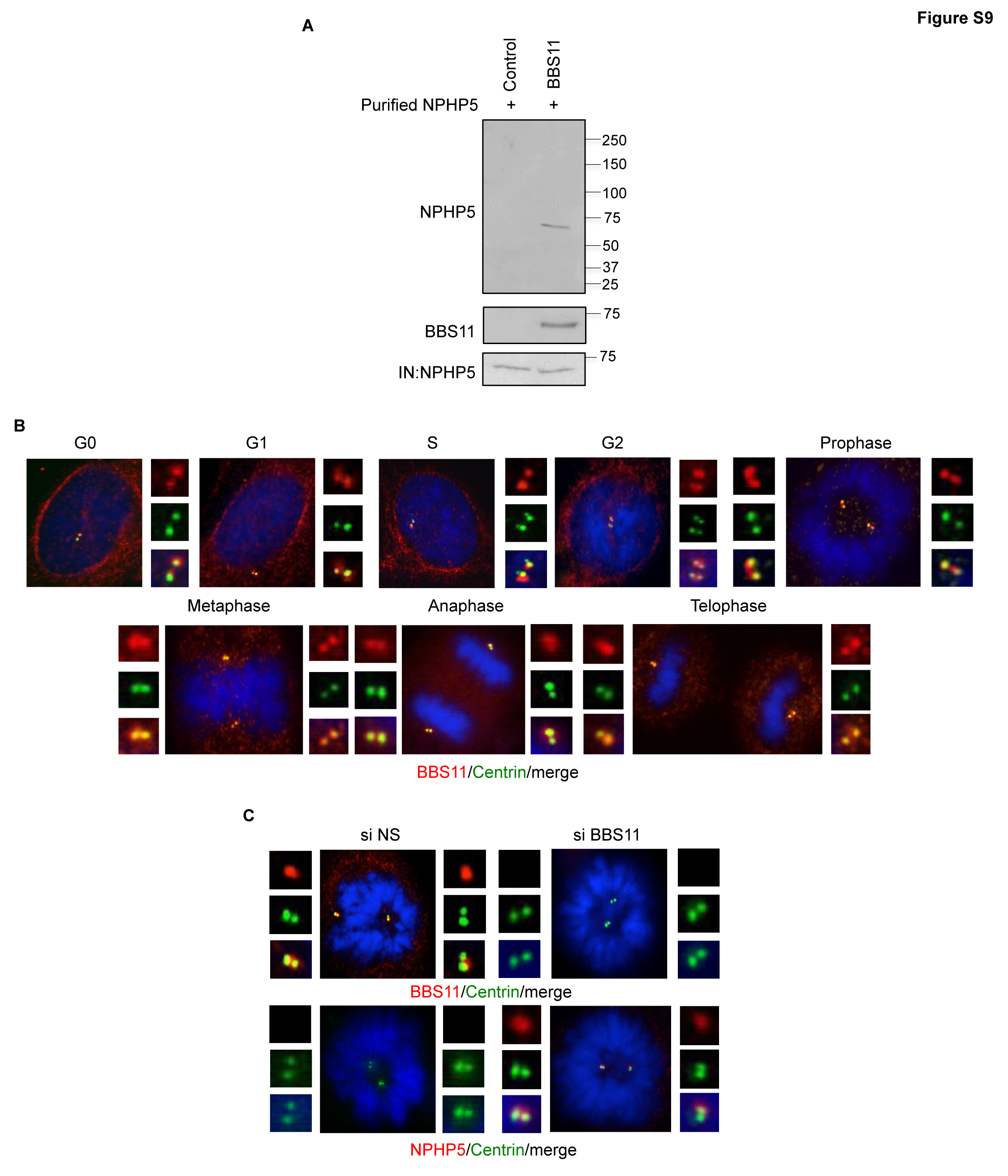

Supplement: S9 Fig — A) HEK293 cell lysates were immunoprecipitated with anti-IgG (control) or anti-BBS11 antibody. Endogenous BBS11 bound to beads was then incubated with purified Flag-NPHP5. Proteins recovered on beads were analyzed by Western blotting with the indicated antibodies. IN, input. B) RPE-1 cells in different stages of the cell cycle were processed for immunofluorescence with BBS11 (red) and centrin (green) antibodies. DNA was stained with DAPI (blue). C) Cycling RPE-1 cells were transfected with control (NS) or BBS11 siRNA and stained with antibodies against BBS11 or NPHP5 (red) and centrin (green). DNA was stained with DAPI (blue). Prophase cells are shown. (TIF) [file pgen.1006791.s009.tif]

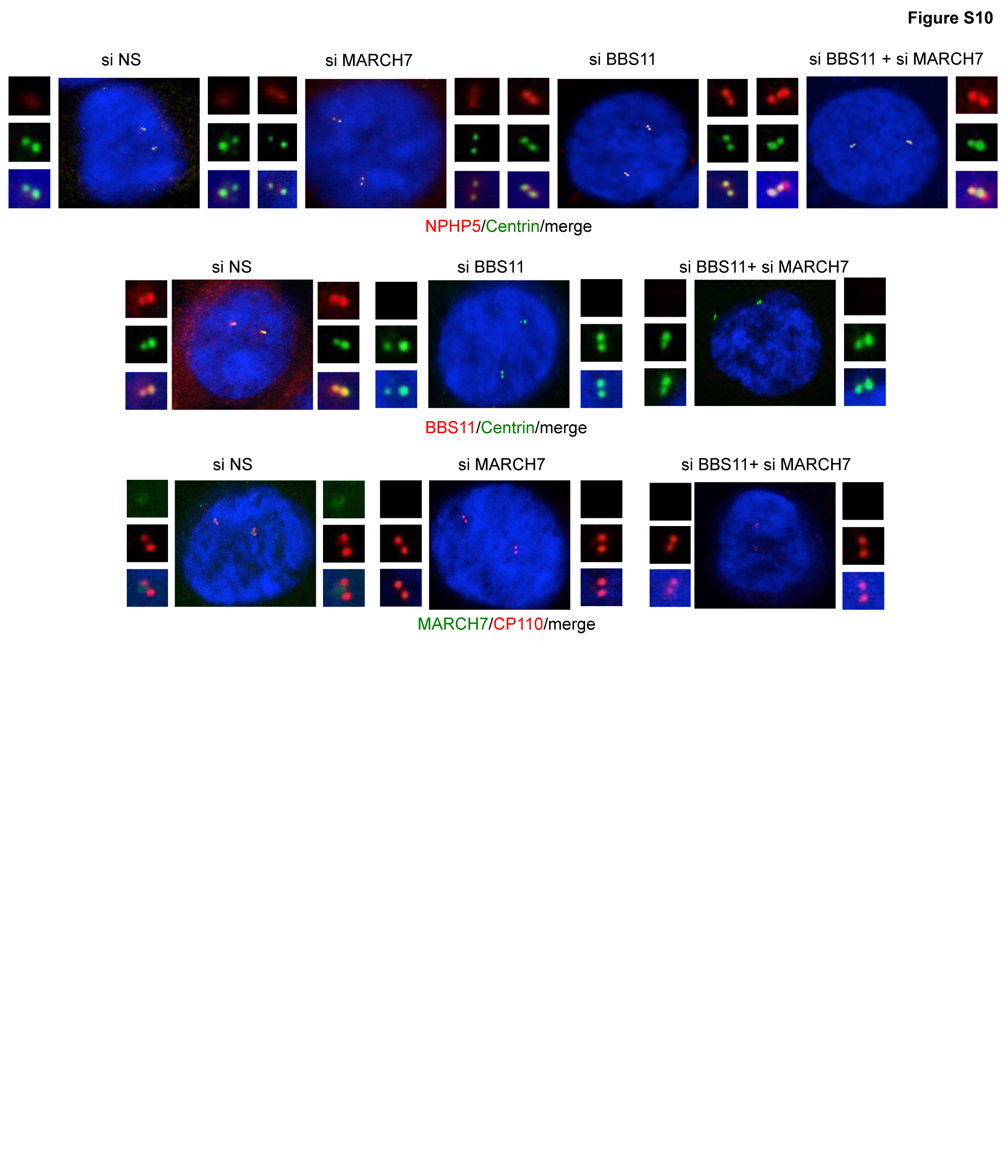

Supplement: S10 Fig — Cycling RPE-1 cells were transfected with control (NS), BBS11, MARCH7 or BBS11 and MARCH7 siRNA and stained with antibodies against BBS11, CP110 or NPHP5 (red) and centrin or MARCH7 (green). DNA was stained with DAPI (blue). Late G2 cells are shown. (TIF) [file pgen.1006791.s010.tif]

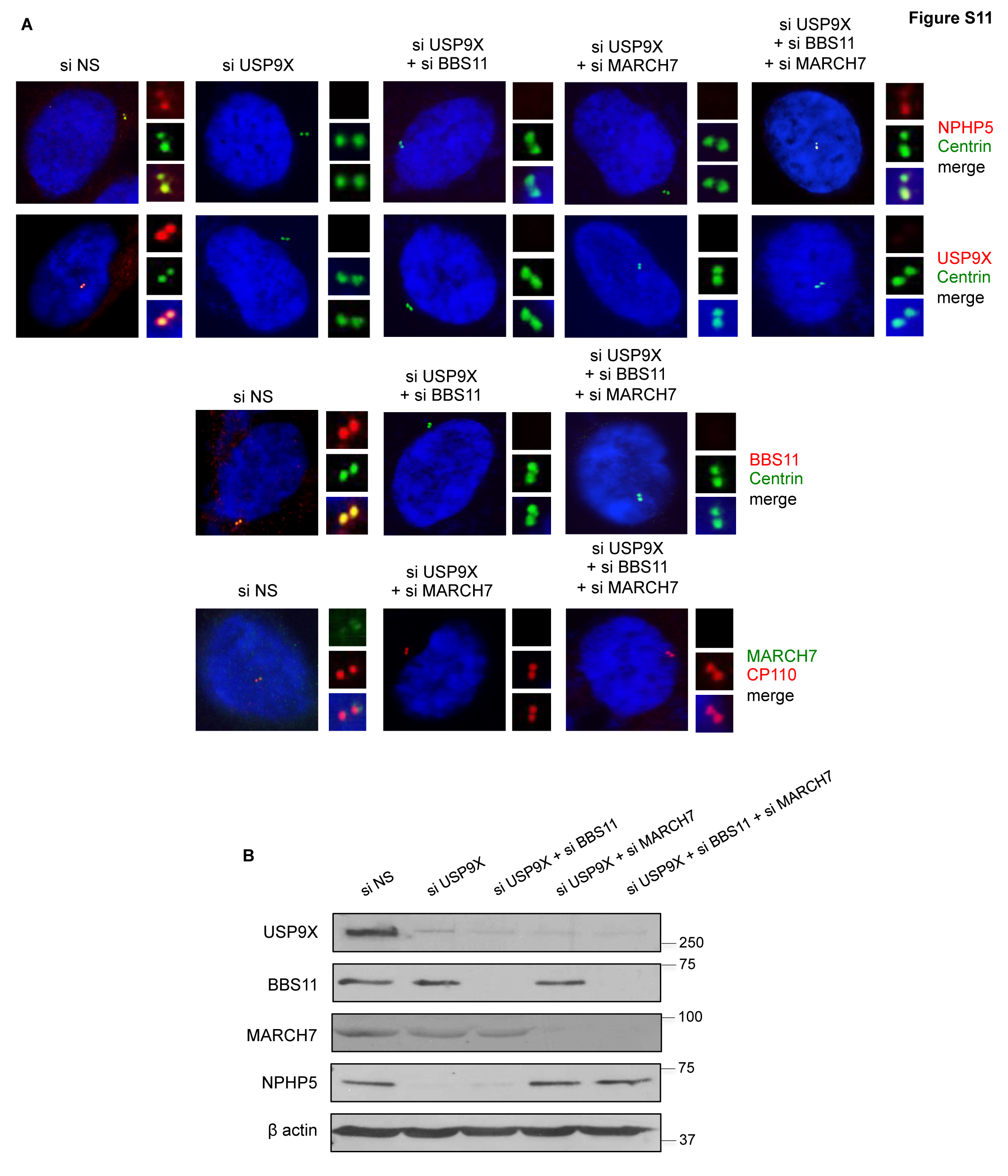

Supplement: S11 Fig — A) RPE-1 cells transfected with control (NS), USP9X, or USP9X in combination with MARCH7 and/or BBS11 siRNA, and stained with antibodies against USP9X, NPHP5, BBS11 or CP110 (red) and MARCH7 or centrin (green). DNA was stained with DAPI (blue). B) HEK293 cells were transfected with control (NS), USP9X, or USP9X in combination with MARCH7 and/or BBS11 siRNA, Lysates were Western blotted with the indicated antibodies. β actin was used as a loading control. (TIF) [file pgen.1006791.s011.tif]
